# Reshaping of a Glycoside Hydrolase Active Site through Expression-Compensated Droplet-Based Microfluidic Screening Provides Useful Tools for Glycomics

**DOI:** 10.1021/acscentsci.5c01227

**Published:** 2025-09-02

**Authors:** Jacob F. Wardman, Feng Liu, Saulius Vainauskas, Charlotte Olagnon, Teresa A. Howard, Yuqing Tian, Seyed A. Nasseri, Rajneesh K. Bains, Christopher H. Taron, Stephen G. Withers

**Affiliations:** † Department of Biochemistry and Molecular Biology, 8166 University of British Columbia, Vancouver, BC V6T 1Z3, Canada; ‡ Michael Smith Laboratories, University of British Columbia, Vancouver, BC V6T 1Z4, Canada; § Department of Chemistry, University of British Columbia, Vancouver, BC V6T 1Z1, Canada; ∥ 1696 New England Biolabs, Ipswich, Massachusetts 01938, United States

## Abstract

The glycosylation of proteins endows them with distinct biophysical properties and allows them to play fundamental roles in cellular communication. Much of our understanding of glycoproteins has derived from the ability to enzymatically manipulate glycan structures. In particular, selective cleavage of glycans from proteins simplifies the analysis of glycoproteins and the determination of structure–activity relationships. However, limited enzymatic tools are available for the study of mucin-type O-glycans. To address this, we carried out the directed evolution of a glycoside hydrolase to increase its ability to cleave the sialyl T-antigen, a ubiquitous O-glycan structure in humans. We employed ultrahigh-throughput droplet-based microfluidics to rapidly screen vast libraries of variants in pL-sized droplets, thus minimizing the quantities of complex substrate required. Furthermore, by use of fluorescent protein-fusion and ratiometric gating during droplet sorting we could account for varying expression levels and identify highly active hits that could have been overlooked due to lower expression levels. Within just two rounds of screening, we uncovered variants with 840-fold enhancements in activity and new specificities compared to those of the WT enzyme. This campaign highlights the versatility of glycoside hydrolases and provides a broadly applicable strategy to engineer enzymatic tools for glycomics through microfluidic screening.

## Graphical abstract

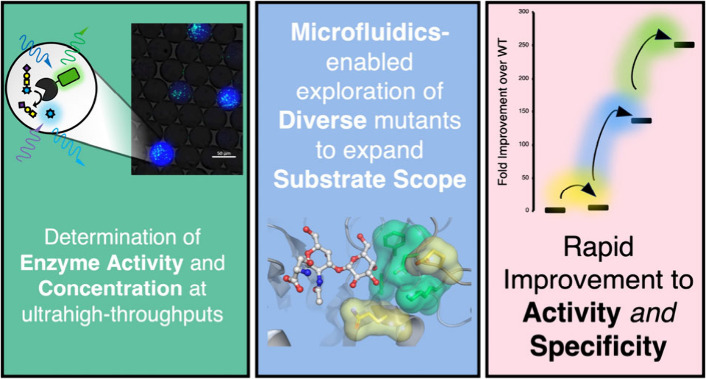

## Introduction

At the cell surface, carbohydrates (or glycans) are often conjugated to proteins to form glycoproteins, where they can play important roles in cellular communication.^1^ This is owing not just to their location at the cell surface, but also to their potential to carry a large amount of information. The existence of a number of different sugar monomers, each of which can be linked by up to ∼8 different linkages offers the potential for very large numbers of different structures from even small oligosaccharides, far exceeding the possibilities offered by peptides or oligonucleotides of comparable size.^2,3^ Indeed, the different glycan structures found form the so-called “glycocode” which can be read by glycan-binding proteins.^4^ Further complicating the study of glycoproteins is the non-templated biosynthesis of glycans.^1^ The specific glycan structures present on a given glycoprotein sample are dependent upon the properties of the glycosylation site(s) on the protein, the presence of other post-translational modifications, the expression levels of the required glycosyltransferases, and also upon the interplay with metabolism and other factors related to cell type and development.^1,5^ Crucially, differences in both the glycan structure attached to the protein and site(s) of attachment impart different properties to the resultant glycoprotein.^1^

Our ability to understand many aspects of the roles of glycoproteins has been enabled by the availability of enzymatic tools for glycan analysis and manipulation. Enzymatic release of glycans from proteins is commonly performed in order to simplify the samples for mass spectrometry and other analyses.^6^ Enzymes such as peptide-*N*-glycosidase F (PNGase F) or the endo-β-N-acetylglucosaminidases (ENGases)^6,7^ are endo-acting enzymes that catalyze the release of a broad variety of intact N-glycan structures (complete release for PNGase F while leaving a single GlcNAc for ENGases) under gentle conditions.^6,8^ Such released glycans can then be readily characterized by mass spectrometry and/or other analytical methods while leaving the protein largely undisturbed. This further enables downstream characterization of the deglycosylated protein and, through enzymatic glycan remodeling (often with variants of ENGases engineered for glycan synthesis), the roles of specific N-glycoforms can be further probed.^8,9^ The availability of these well-established workflows has enabled in-depth study of glycoform structure–activity relationships for N-glycans, such as the roles of different antibody glycan structures in effecting antibody-dependent cellular cytotoxicity.^8,10,11^

Mucin-type O-glycans (hereafter referred to as O-glycans), are another common form of glycosylation in animals.^12^ Indeed, recent work has suggested that 83% of secreted proteins can be O-glycosylated.^13^ O-Glycosylation is defined by the initial attachment of *N*-acetylgalactosamine by an α-linkage to serine, threonine and, in certain circumstances, tyrosine residues.^12,14^ In recent years, there has been an increased appreciation of the roles of mucins and O-glycans in interactions with our microbiome,^15^ in controlling the activities and half-lives of circulating peptides and proteins,^12,14,16^ as well as in different aspects of immunomodulation by cancers.^17,18^

Despite their importance, there are essentially no equivalents of PNGase F or ENGases for the universal enzymatic release of O-glycans from the protein backbone. Thus, making analysis and engineering of O-glycoproteins more challenging. Besides broadly acting O-glycanases, identification of highly specific enzymes—capable of cleaving only select glycan structures—would also be of great use for deciphering the roles of specific glycan structures.

The closest enzymatic equivalents to ENGases or PNGase for O-glycans are a family of endo-α-GalNAcases from glycoside hydrolase family 101 (GH101).^6,19^ Unfortunately, GH101s only cleave a very limited subset of O-glycan structures—making study of most O-glycans inaccessible by these methods.^19^ As an example, the native substrate of most GH101s is the disaccharide T-antigen (Gal-β1,3GalNAc-α1-Ser/Thr). This structure is relatively rare in healthy adults and instead is often associated with cancer cells.^20^

A number of campaigns have explored the natural diversity of glycoenzymes in attempts to identify new and improved catalysts for different purposes.^21^ In our previous work,^22^ we used functional metagenomic screening of a human gut microbiome library to search for enzymes that cleave off one of the most common O-glycans in humans, the sialyl T-antigen (STAg) (Neu5Ac-α2,3Gal-β1,3GalNAc-α1-Ser/Thr).^23–25^ In that work we discovered that GH101s, which were thought to be entirely unable to cleave off STAg, actually catalyzed the reaction, but quite slowly and with a strong preference (>1000-fold) for their native substrate, the T-antigen.^22^ In fact we were somewhat surprised that we had not identified more enzymes in that screen since that same library has, to date, yielded five distinct A blood type antigen-cleaving enzymes/enzyme systems,^26^ numerous exo-β-GlcNAcases,^27^ unsaturated β-glucuronidases,^28^ a B antigen cleaving enzyme in a number of different contexts,^29^ and gene clusters encoding broadly specific enzymes capable of the stepwise breakdown of a number of carbohydrates via an unconventional mechanism.^30^ We therefore sought other avenues to access practical enzymes for O-glycan analysis.

Another popular approach for generating useful enzymes is to select known enzymes with low activity, and then use directed evolution to improve their properties. An excellent example of this is Kwan et al.’s campaign to generate broad-specificity blood group-cleaving GH98 endoglycosidases.^31^ In that work, over five rounds of screening, they were able to improve activity 170-fold against a blood group antigen with a non-preferred linkage.^31^ However, microtiter plate screens, as employed in that work, require substantial amounts of substrate (typically multimilligram amounts per plate), and handling steps that limit typical throughputs to <10^5^/day; with many screens often only surveying 100s-1000s of clones/round.^32^

Directed evolution combined with ultrahigh-throughput screening via droplet-based microfluidics has proven to be an especially potent approach for improving enzymes.^33^ In these microfluidic assays, an oil emulsion serves to compartmentalize the reactions in much the same way as the wells of a microtiter plate. However, the pL-sized droplets formed offer >10^6^-fold reduction in substrate consumption compared to the μL-scale of a plate-based assay^34^—thus, allowing the use of more complex substrates for which plate-based screening would not be feasible.^29^ Such a capability is especially ideal in developing tools for glycomics as the substrates are often synthetically expensive and only available in limited quantities.^35^ Further, in conjunction with appropriate sorting capabilities, droplet microfluidics allows one to readily screen libraries of >10^5^ clones with ease.^33^ And so, because of the ability to cover vast swathes of sequence space, improvements in enzymatic activity of >100-fold in only one or two rounds of screening are not uncommon in campaigns using these methods.^36,37^

Herein, we employ droplet-based microfluidics in the rapid remodeling of a GH101 active site to effect changes in activity and substrate preference. Given our previous results, we suspected that only a limited number of functional scaffolds for O-glycan cleavage are present in nature. We therefore decided to subject the best mutant from our previous work, *Sp*GH101 Q868G, to directed evolution in order to further improve its activities. As described herein we also employ a fluorescent protein tag to monitor enzyme concentration during droplet and plate screening. This approach enables enrichment of hits with improved activity rather than just improved expression or solubility. By combining these different methods, we were able to identify enzymes with >30-fold enhancements in *k*_cat_/*K*_M_ over the parent enzyme in a single round despite large decreases in expression level. Ultimately, with just two rounds of screening, we identified variants with almost 3 orders of magnitude enhancements in activity over the WT and also 8900-fold changes in selectivity for the STAg over the enzyme’s native substrate. The success of this work demonstrates both the utility of droplet-based microfluidics for efficient exploration of sequence space and the evolvability of GH101s for the selective and practical cleavage of different O-glycan structures.

## Results and Discussion

### Ultrahigh-Throughput Screening with an Expression Reporter for Improved Fidelity

In directed evolution campaigns for improved enzymatic activity, initially promising hits often turn out to have higher expression levels rather than actual improved activity. This is because mutations can also drive changes in stability and or transcription/translation rates, resulting in libraries of mutants having heterogeneous expression levels.^38–40^ For instance, a deep mutational scanning study on the cytochrome P450, CYPC29, demonstrated that within a pool of point mutants, almost half of the variations in observed enzyme activity can be explained entirely by differences in protein abundance and stability.^39^ While improved stability and/or expression is often a desirable property, it is separate from the goal of many directed evolution efforts, whose main aim is to improve enzymatic activity. A number of different approaches to account for these confounding factors have been implemented during directed evolution campaigns. These have included appending of short split GFP tags,^38^ of epitope tags that can be detected by antibodies,^41^ or the direct fusion of whole fluorescent proteins.^38,42,43^ Owing to difficulties in droplet manipulation, this latter approach is the most readily compatible with droplet-based microfluidics. A further benefit of this approach is that often the fluorescent protein domain can only fold correctly (and thus produce a fluorescent signal) if the attached protein of interest is also folded.^44^ And so, for this work, we fused the fluorescent protein mNeonGreen to the C-terminus of *Sp*GH101 Q868G.^45^ The mNeonGreen protein is ideal for such a purpose because of its brightness (and thus high sensitivity) and fast maturation time which is necessary for accurate measurement of protein concentration.^43,45^ As well, given the spectral separation between mNeonGreen (EX: 506 nm, EM: 517 nm)^45^ and the Jericho Blue fluorophore which we use to measure activity in our screen (further detailed below) (EX: 390 nm, EM: 450 nm),^46^ both enzyme concentration and activity can be read simultaneously in both flow cytometry and microtiter plate assays, with limited interference (Figure 1A).

**Figure 1:**
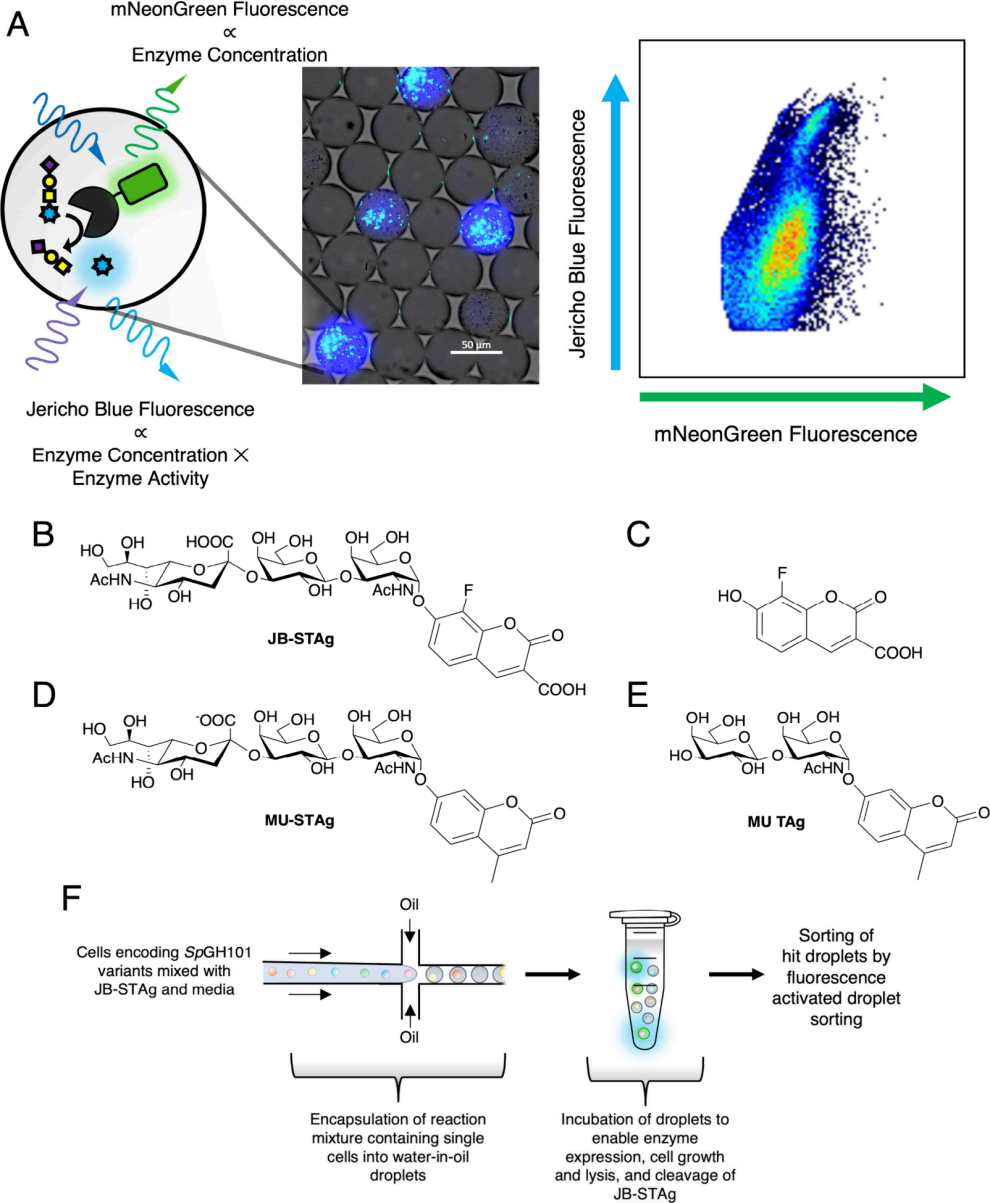
**Strategy and substrates used to screen for improved STAg hydrolases. A**, Measurement of enzyme concentration via an appended mNeonGreen tag and substrate cleavage via Jericho Blue fluorescence provides a more accurate picture of enzyme activity. Central panel shows the droplets during one of the rounds of sorting in this study while the right panel shows the results of droplet analysis by flow cytometry. **B**, The Jericho Blue sialyl T-antigen substrate used for screening, **C**, the Jericho Blue fluorophore, **D**, the methylumbelliferyl sialyl T-antigen (MU-STAg), and **E**, the methylumbelliferyl T-antigen (MU-TAg) used for kinetic characterization in this study. **F**, Workflow for screening of *Sp*GH101 variants by droplet-based microfluidics.

Previous work has shown that enzymes often have lower activity when the fluorescent protein is appended.^38,42,43^ To see if this was also the case in our construct, we expressed and then measured the activity of the *Sp*GH101 Q868G mNeonGreen fusion protein in the clarified lysate, using the mNeonGreen tag for quantification. Gratifyingly, we were able to measure a *k*_cat_/*K*_M_ value against MU-TAg (Gal-β1,3GalNAc-α1-MU) (3300 ± 100 s^–1^ mM^–1^) that is comparable to that which we had previously measured with purified nontagged enzyme (2700 ± 200 s^–1^ mM^–1^).^22^ It is possible that *Sp*GH101 is more accepting of the fused mNeonGreen domain than literature examples as it is naturally a multidomain protein with several appended carbohydrate binding modules.^47^ In addition, as a consequence of the presence of these additional domains, mNeonGreen is well separated from the enzyme’s catalytic domain, minimizing the risk of interference in catalytic activity.

For our directed evolution campaign, we employed chemoenzymatic synthesis to prepare a substrate consisting of STAg attached to a fluorophore known as Jericho Blue (Neu5Ac-α2,3-Gal-β1,3-GalNAc-α1-JB) (JB-STAg) (Figure 1B). Jericho Blue is a fluorophore developed by the Withers laboratory to obviate the problem of leakage of the fluorophore during droplet microfluidic screening (Figure 1C). Depending on the oil used, free fluorophore can either diffuse freely into the bulk oil or undergo vesicle-mediated transport between droplets.^32,48^ Such effects break the genotype-phenotype linkage and are thus undesirable for screening. Indeed, these effects makes use of substrates such as methylumbelliferyl sialyl T-antigen (MU-STAg)—which we had previously used in our plate-based screening and kinetic characterization of GH101s^22^—inappropriate for screening in droplets (Figure 1D). The most straightforward approach to ablating such leakage is to introduce charge onto the fluorophore.^33^ Besides the inclusion of a negatively charged carboxylate on the coumarin core for droplet retention, inclusion of a fluorine in the phenolic moiety of the fluorophore renders it more acidic (p*K*_a_ = 6.0). This low p*K*_a_ ensures that the majority of the fluorophore is in its deprotonated, fluorescent phenolate form at pH 7.0.^46^ This increases the sensitivity at neutral pH, avoiding any need to carry out the screen at a high pH or use of a stopped reaction in order to improve sensitivity.^46^

Having established a means to accurately measure enzyme activity and concentration, as well as a suitable substrate, we proceeded into droplet-based microfluidic screening of a library of *Sp*GH101 Q868G mutants for enhanced activity against JB-STAg (Figure 1F). In this scheme, live cells are encapsulated within the droplets along with media and the JB-STAg substrate. Over the course of growth, a certain number of cells within the droplet will naturally lyse, releasing the expressed *Sp*GH101 variant, and allowing the enzyme to interact with its substrate. The droplets are then sorted, and live cells are recovered from the droplets. These cells can then be grown again for additional rounds of droplet-based sorting or arrayed into microtiter plates for validation. This relatively straightforward approach has been used in other studies^29,34^ and avoids the difficulty in lysing cells within the droplet or in having to recover DNA after sorting.

We generated a library of 60,000 mutants containing 1–8 mutations per gene by error-prone PCR (epPCR). The library was then subjected to four rounds of sorting for activity against JB-STAg (Figure 2A). The sorting gates were set to capture those droplets with high activity against JB-STAg (blue fluorescence) that are present at moderate enzyme concentration based upon mNeonGreen fluorescence (see Figure S1 for an example gating strategy). Initial attempts to capture active droplets expressing only low enzyme concentrations, which without enzyme quantification would be otherwise lost, resulted largely in isolation of clones with a stop codon prior to the fused mNeonGreen and so were not pursued. Across the four rounds of sorting there was a clear increase in the level of Jericho Blue released within the droplets (Figure 2A). In the final round of sorting, the Jericho Blue fluorescence likely exceeded the linear range of the assay resulting in a sharp asymmetrical peak.

**Figure 2:**
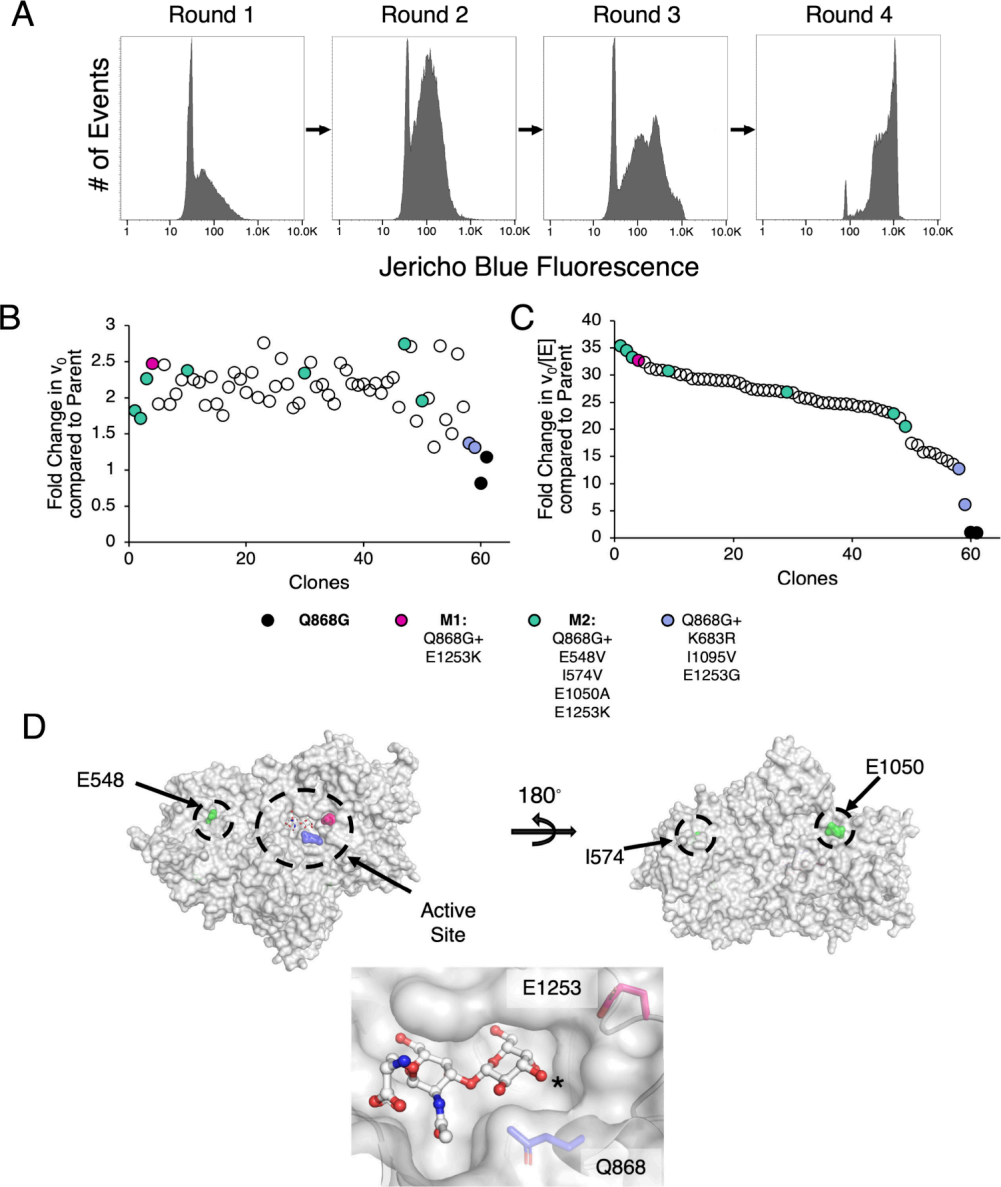
**Screening an epPCR library for improved STAg hydrolases in droplets provides rapid improvement. A**, Increase in free Jericho Blue fluorescence from enrichment of improved JB-STAg hydrolases across rounds of screening in droplets. Plate-based validation of droplet screening without (panel **B**) and with normalization (panel **C**) for enzyme concentration based on mNeonGreen fluorescence. Black data points indicate parent (*Sp*GH101 Q868G) controls. **D**, Location of mutations found in the hits, M1 and M2, derived from screening an epPCR library (PDB ID: 5A58).^53,54^ The lower panel shows the active site with serinyl-TAg bound and highlights the locations of the parental Q868G mutation and the highly influential E1253 residue. Note that both M1 and M2 share the E1253K mutation while another improved mutant was found to contain the mutation E1253G. The asterisk indicates the C3–OH where Neu5Ac would be appended in the STAg.

Subsequent plate-based screening of cells recovered from the droplets after the fourth round of sorting revealed substantial enrichment of clones with highly enhanced activity. Enzymes in the recovered clones were expressed, the cells lysed and then assayed for activity. Enzyme concentrations were determined by mNeonGreen fluorescence while the enzymatic activity was determined via cleavage of JB-STAg. 59 of the 94 recovered clones showed relatively modest improvement (up to 2.7-fold) compared to the parent on the basis of observed v_0_ (Figure 2B). However, when enzyme concentrations were taken into account, an average of a 25-fold improvement with a maximum of 35-fold improvement was observed (Figure 2C). Consistent with this, and the fact that overall activities were only slightly higher, both the mNeonGreen fluorescence and SDS-PAGE analysis confirmed that the expression levels of these mutants were much lower compared to the parent (∼10-fold lower upon the basis of mNeonGreen fluorescence) (Figure S2).

### Mutational Analysis of Hit Variants Demonstrates the Critical Role of E1253 in STAg Hydrolysis

The top four mutants, along with a selection of others with differing levels of activity, were recovered and sequenced (Figure 2C). Of the top four, one mutant (M1) was found to contain a single point mutation (E1253K) in addition to the parental Q868G while the remaining three were identical (M2) and contained Q868G, E1253K as well as three additional mutations (E548V, I574V, and E1050A). The majority of the clones sequenced, even those with apparently different activity levels, turned out to be the M2 variant. However, those with the lowest levels of improvement were found to contain the mutation E1253G in addition to two others (K683R, I1095V) (Figure 2C). Given the lower activity of this mutant, we proceeded forward with characterization of only the M1 and M2 variants. However, such results do suggest that the E1253 position is important in determining the activity of GH101s against the STAg.

Kinetic characterization of purified M1 and M2 revealed a 20- and 29-fold improvement in MU-STAg activity compared to the parent Q868G (Table 1). In the case of M2 this amounts to a 138-fold improvement compared to the WT and a very respectable *k*_cat_/*K*_M_ of 40 ± 2 s^–1^ mM^–1^. Deconvolution of the effects of the three remaining mutations in M2 revealed that E548V provided the largest improvement over M1 (∼30%) with I574V and E1050A providing more modest enhancements (∼20% each) (Table S1). Curious as to why these mutants had such low expression levels compared to *Sp*GH101 Q868G, we also measured their thermal stabilities to see if decreased stability played a role. Notably, we observed a modest but significant decrease in the *T*_50_^10^ (that is, the temperature at which after 10 min of incubation, half of the maximal enzyme activity is present) of M1 and M2 compared to *Sp*GH101 Q868G (from 46 to 42 °C and 43 °C respectively) (Table S2). Concerned by this decreased stability, we also measured activity of these mutants against MU-STAg at room temperature and, reassuringly, found comparable levels of improvement (30-fold) to those seen at 37 °C (Table 2).

**Table 1:** Kinetic Parameters for Cleavage of MU-STAg and MU-TAg by Evolved *Sp*GH101 Variants at 37 °C^a^

	MU-STAg		MU-TAg				MU-TAg/MU-STAg
*Sp*GH101 Mutant	*k*_cat_/*K*_M_ (s^–1^ mM^–1^)	Fold-Change in *k*_cat_/*K*_M_ over WT	*k*_cat_ (s^–1^)	*K*_M_ (μM)	*k*_cat_/*K*_M_ (s^–1^ mM^–1^)	Fold-Change in *k*_cat_/*K*_M_ over WT	*k*_cat_/*K*_M_
**WT** ^b^	0.29 ± 0.01^c^	1	70 ± 5	79 ± 11	900 ± 100	1	3103
**Q868G** ^b^	1.4 ± 0.1^c^	4.8	366 ± 9	135 ± 9	2700 ± 200	3	1929
**M1**	27.2 ± 1.4^c^	94	105 ± 8	530 ± 105	198 ± 42	0.22	7.3
Q868G							
E1253K							
**M2**	40 ± 2^c^	138	52 ± 2	351 ± 45	147 ± 20	0.16	3.7
E548V							
I574V							
Q868G							
E1050A							
E1253K							
**M3**	73 ± 3.7^c^	252	107 ± 9	240 ± 65	444 ± 126	0.49	6.1
E548V							
I574V							
Q868G							
E1050A							
K1156S							
E1253K							
**M4**	22 ± 1^c^	76	ND	ND	7.8 ± 0.4^c^	0.009^c^	0.35^c^
E548V							
I574V							
M616F							
Q868G							
E1050A							
K1156L							
E1253K							
D1254N							

a All values are shown as mean ± standard error of the mean (*n* = 3 or 4). An error of 5% was assumed for all values unless determined to be higher experimentally. ND indicates not determined.

b From previous work.^22^

c The GH101s assayed did not display saturation kinetics toward the substrate, and so the substrate depletion method was used to determine *k*_cat_/*K*_M_ as previously described.^55^ Please see the Methods section for additional details on substrate depletion kinetics.

**Table 2:** Kinetic Parameters for Cleavage of MU-STAg by Evolved *Sp*GH101 Variants at Room Temperature^a^

*Sp*GH101 Mutant	*k*_cat_/*K*_M_ (s^–1^ mM^–1^)	Fold-Change in *k*_cat/_ *K*_M_ over WT
WT	0.068 ± 0.003	1
Q868G	0.46 ± 0.02	6.8
M1	14.4 ± 0.7	212
M2	14.5 ± 0.7	213
M3	57 ± 3	838
M4	9.8 ± 0.5	144

a All values are shown as mean ± standard error of the mean (*n* = 3). An error of 5% was assumed for all values unless determined to be higher experimentally. The GH101s assayed did not display saturation kinetics toward MU-STAg, and so the substrate depletion method was used to determine *k*_cat_/*K*_M_ as previously described.^55^

Based upon the improvement observed in M1, it seemed that the E1253K mutation is the main driver of the increase in activity. Examination of the crystal structure of *Sp*GH101 TIGR4 (which shows 98.8% similarity to the *Sp*GH101 R6 variant that was used as the template for this study) with bound serinyl TAg shows that E1253 sits at the edge of the −2 subsite near C3/C4 of Gal (Figure 2D). This would put it in close proximity to the 2,3-linked sialic acid of the STAg. The conversion of electrostatic repulsion to attraction is an appealing explanation for this increase in activity. In a broad sense, many analogous charge-accommodating mutations have been identified in the evolution and engineering of other enzymes for activity against sulfated and sialylated glycans.^49–51^ As well, a number of mutations to the residue equivalent to E1253 have been shown to introduce STAg hydrolase activity into the GH101 EngBF from *Bifidobacterium longum*.^52^ In that work, molecular docking was used to guide the introduction of mutations that enabled STAg-hydrolase activity in EngBF.^52^ It is remarkable that these diverse techniques can independently converge upon such mutations. However, the activity of these EngBF mutants still appears to be >1000-fold lower against STAg as compared to TAg when using fetuin as a substrate.^52^ By comparison, the differences in activity between the MU-TAg and MU-STAg substrates seen with *Sp*GH101 M1 and M2 are much more modest (Table 1).

Previously we had observed a notable disconnect between the level of activity exhibited by enzymes of CAZy family GH101s in the cleavage of chromogenic substrates versus that with correspondingly substituted glycoproteins.^22^ To check if such was the case here also, we performed time course assays to compare the rate of STAg release from fetuin using the improved mutants, with that of *Sp*GH101 Q868G. Satisfyingly, we observed large increases in activity in all cases (Figure S3a). Remarkably, M1 and M2 appear to cleave STAg from fetuin 13- and 20-fold more quickly than does *Sp*GH101 Q868G. This is largely in line with the improvement in *k*_cat_/*K*_M_ against MU-STAg (Table 1). Additional experiments measured similar activity changes across all other mutants as well (Figure S3b) confirming that, at least for *Sp*GH101, activities on chromogenic substrates and proteins correlate very well.

We further compared the activity of our M2 variant against the widely used O-glycosidase from New England Biolabs using high sensitivity UPLC-FLR-MS (Figure 3). Analysis of activity against fetuin showed release of a number of glycans by M2 that could not be cleaved by a commercial O-glycosidase (Figure 3A). These include species corresponding to the STAg but also to HexNAc(1)­Hex(1)­NeuGc(1) (Figure 3B). The therapeutic etanercept was also analyzed by this method (Figure 3C). Again, we observed clear release of STAg by *Sp*GH101 M2 but not by the commercial O-glycosidase.

**Figure 3:**
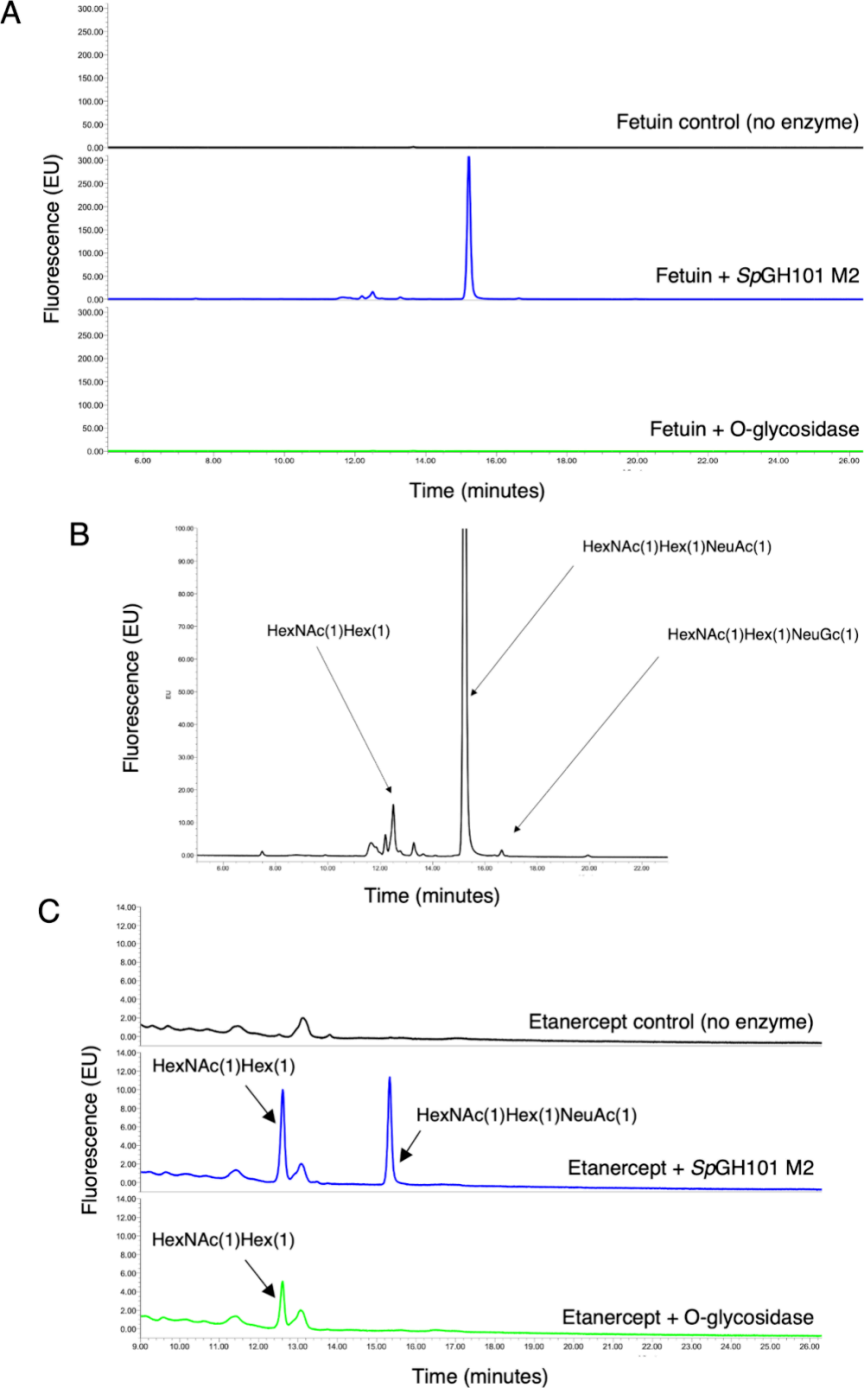
**Activity of *Sp*GH101 M2 against fetuin and etanercept as compared to commercially available O-glycosidase.** Protein samples were incubated with either *Sp*GH101 M2 or NEB O-glycosidase overnight at 37 °C. The released glycans were then labeled with procainamide and analyzed by UPLC-FLR-MS. **A**, Activity of the enzymes against fetuin. **B**, Peak assignments for the *Sp*GH101 M2 released glycans. **C**, Activity of the enzymes against etanercept.

### Combinatorial Site-Saturation Mutagenesis Yields Further Improvements and New Substrate Preferences

Given the key roles of active site residues (Q868 and E1253) in the improvements to *Sp*GH101 thus far, we decided to focus our efforts to further improve the enzyme through mutating additional residues surrounding the putative −3 subsite (Figure 4A). We generated a combinatorial library of mutants in which two largely buried residues M616 and F618, were simultaneously mutated to different hydrophobic amino acids while the more exposed K1156 and D1254 were simultaneously subjected to complete site saturation. Based upon the scheme used, the generated library could maximally contain 43,008 different variants at the DNA level and 12,000 unique protein variants. This diversity was well covered by a newly generated library containing 330,000 clones. Given the high activities of the M2 parent, which exceeded the range of the assay in the previous round of screening, we conducted the droplet screening at a lower temperature (30 °C as compared to 37 °C in the initial screen) and did not include IPTG to induce expression, instead relying on leaky expression. After two rounds of screening in droplets we then assayed 186 of the recovered clones in two 96-well plates for activity against JB-STAg and MU-TAg. As shown in Figure 4b and Figure S4, this library contained mutants of diverse activities with differing preferences toward the STAg and TAg substrates. Sequencing of different variants further revealed a variety of different combinations of mutations which conferred these activities. Despite these differences in activities, we did not observe the same losses in stability/expression as observed in the previous round of screening (Figure S5). This may be due to reaching a lower limit in terms of the stability of the enzymes and/or the amount of active enzyme required for detection in our system.

**Figure 4:**
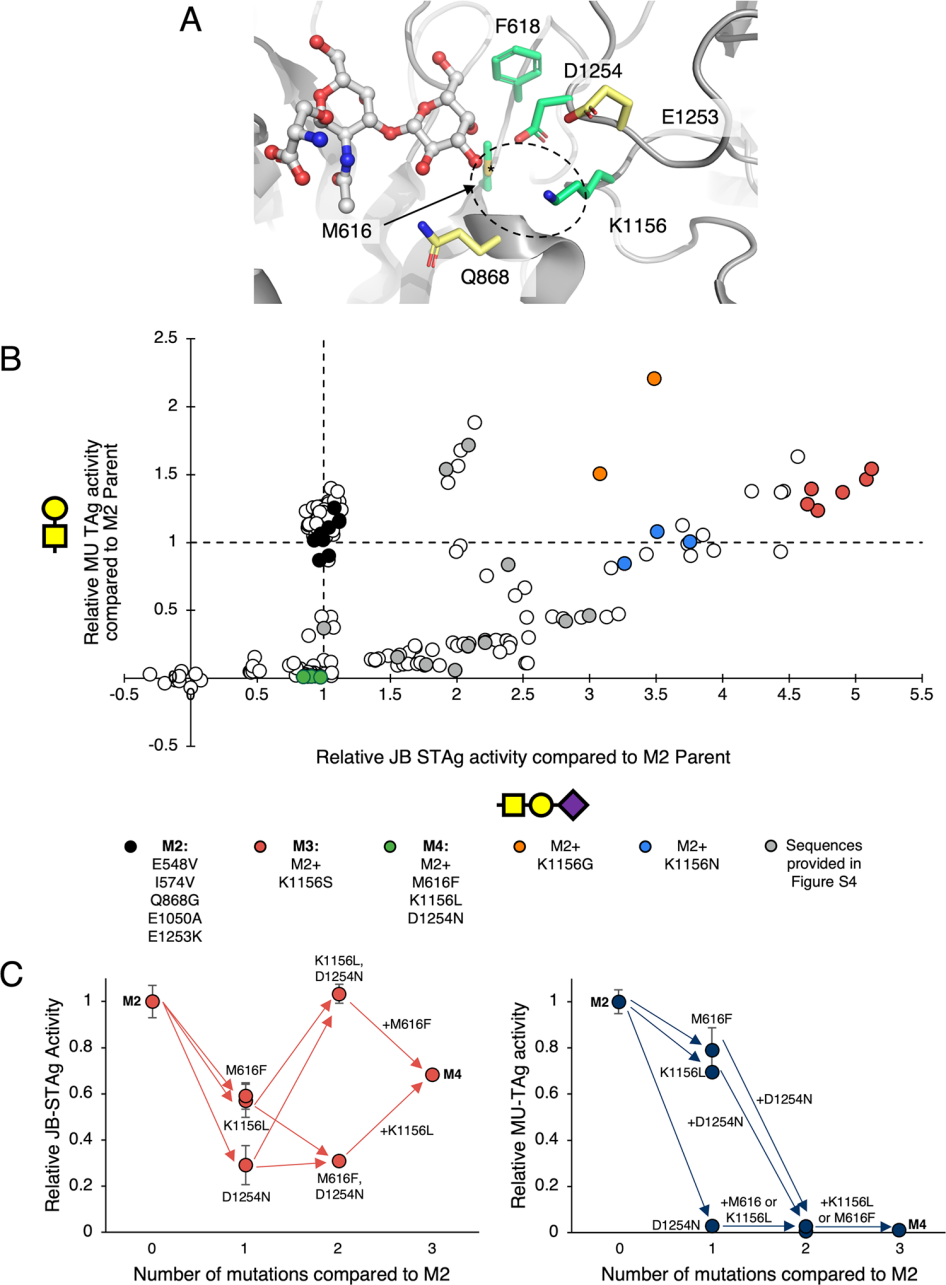
**Simultaneous site-saturation mutagenesis of active site residues produces enzymes of diverse activities. A**, Active site residues surrounding the putative −3 subsite selected for site-saturation mutagenesis (PDB ID: 5A58).^53,54^ K1156 and D1254 were subject to complete site-saturation, while M616 and F618 were only mutated to other hydrophobic residues. The resultant library was then subjected to screening in droplets for activity against JB-STAg. **B**, The variants recovered after two rounds of screening in droplets were then assayed against JB-STAg and MU-TAg in microtiter plates. Further details on the identities of the mutants identified in this screen (shown in gray) are available in Figure S4. White data points were not sequenced. **C**, Screening of lysates from a shuffled library show that epistatic interactions between mutants of M616, F618, and K1156 result in enzymes that are selective for STAg over TAg. Error bars indicate standard error (*n* = 2–6 depending upon the mutant). Only one M616F, D1254N mutant was identified and so no error bars are shown.

Of particular interest was M3 which showed the highest level of JB-STAg activity among the variants and consists of only a single additional point mutation (K1156S) (Figure 4B). Generally, it seems that the identity of the residue at the K1156 position is the strongest determinant of mutant activity against STAg in this library. All nonparent sequenced variants of higher activity contained a mutation at this position, with increases in activity also being observed upon its mutation to Gly or Asn (Figure 4B). Detailed kinetic characterization of purified M3 demonstrated that the enzyme cleaved MU-STAg 1.8-fold faster than M2 and 250-fold faster than the WT enzyme at 37 °C (Table 1). Notably, when activity was measured at room temperature, we observed a 3.9-fold and 838-fold improvement compared to M2 and the WT respectively. We further measured the thermal stability of the mutant and found that M2 and M3 were comparable, suggesting that this difference is due to increased flexibility within the active site rather than any thermal denaturation (Table S2).

The presence of additional mutations at M616, F618 or D1254 seems to provide modest attenuations to the STAg activity with strong effects on selectivity due to much larger effects on TAg activity (Figure 4b and Figure S4). Of particular note is the M4 mutant (M616F, K1156L, D1254N) which, while largely maintaining the STAg activity of the M2 parent, appeared to have completely lost its activity against the TAg (Figure 4B). Kinetic characterization of M4 confirmed the results from the screen. While only a modest decrease in MU-STAg activity was observed there was a drastic 19-fold decrease in MU-TAg activity compared to M2 (or 115-fold compared to WT) (Table 1). Moreover, while the WT enzyme carries a > 3000-fold preference for the MU-TAg this preference is inverted in M4 such that the enzyme actually has a ∼3-fold preference for MU-STAg; amounting to an overall 8900-fold change in selectivity.

To further determine the effects from each of the mutations on M4’s activity compared to M2, we generated a new library by shuffling the M616F, K1156L, and D1254N mutations with the WT residue at each position and then screened the library for activity (Figure S6). Introduction of the M616F or K1156L point mutations resulted in only slight decreases in activity against both MU-STAg and MU-TAg (Figure 4C). By contrast, the D1254N mutant suffered a near complete loss of observable enzymatic activity against MU-TAg and a large decrease in activity against MU-STAg. In a clear case of epistasis, it is only upon formation of the double mutant (K1156L, D1254N) or the M4 triple mutant that STAg activity is selectively recovered (Figure 4C). This trend seems to also hold true for other combinations of mutations of these residues as we observed similar results with other mutants isolated directly from our droplet screen (Figure S7).

It is perhaps of little surprise that these residues have such complex, cooperative effects on MU-TAg activity. Previous structural characterization of *Sp*GH101 TIGR4 has shown that K1156, E1253, and D1254 form a triad that coordinates a water molecule in the apo form of the enzyme, or the C3- and C4-hydroxyls of the −2 Gal when the TAg is bound (Figure 5A).^53^ This interaction with Gal is made either directly or through an intervening water molecule depending upon the binding mode.^53^ Of course, when STAg is bound the Gal C3–OH has been modified with a sialic acid and so these interactions are of decreased importance for substrate recognition, especially in light of additional interactions formed with the sialic acid moiety.

**Figure 5:**
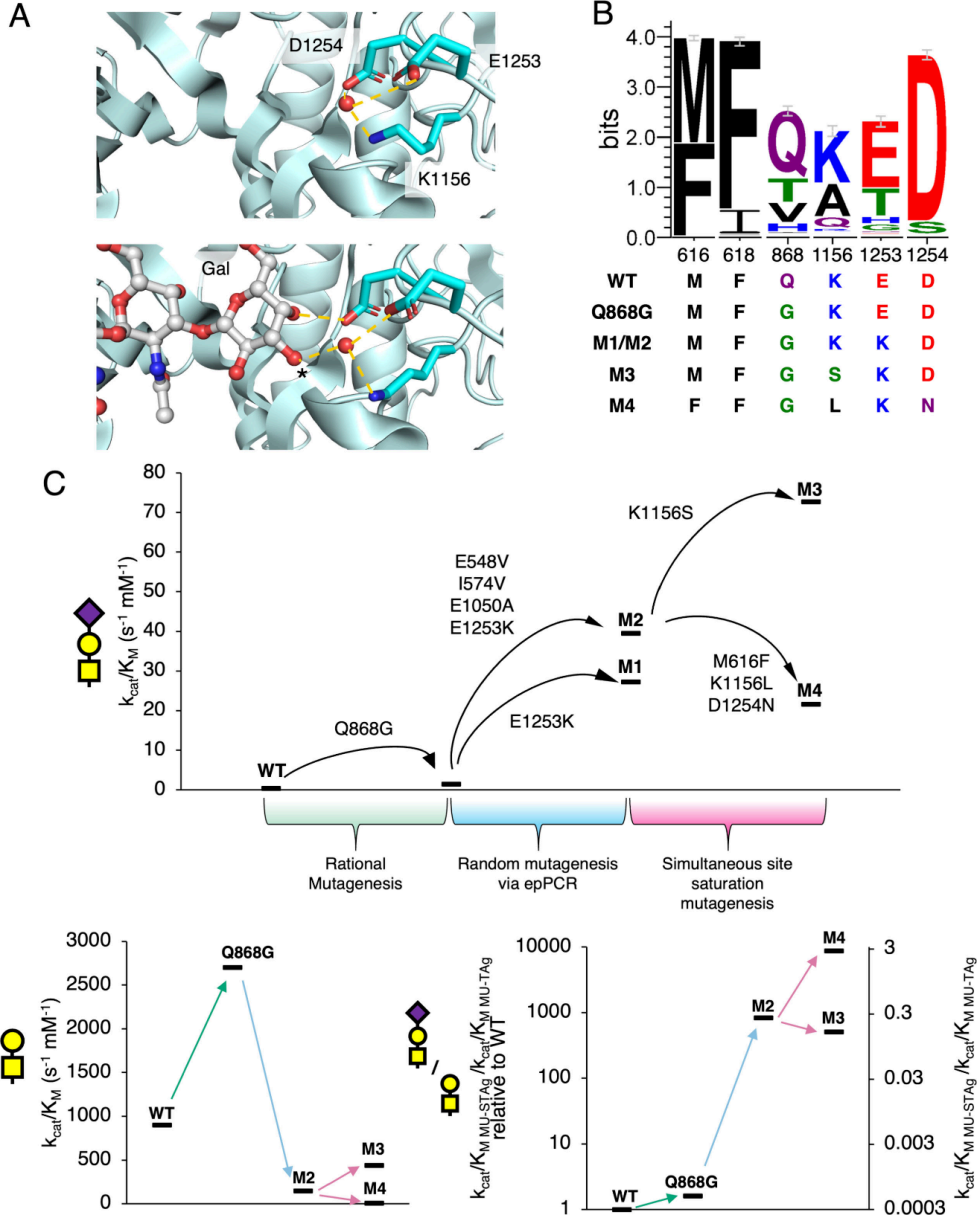
**STAg hydrolases evolved increased specificity and activity throughout this directed evolution campaign. A**, K1156, E1253, and D1254 work together to coordinate a water molecule in the absence of substrate and the C3–OH and C4–OH of the TAg Gal upon substrate binding.^53^ Structures derived from PDB IDs: 5A55 and 5A58.^53,54^ **B**, Sequence logo derived from alignment of all GH101s present in the CAZy database.^74,75^ Note that many of the mutations introduced over the course of directed evolution represent deviations from the consensus. **C**, Evolutionary trajectories of improved *Sp*GH101 STAg hydrolases against MU-STAg, MU-TAg, and the ratio of activity against MU-STAg and MU-TAg. Note that, for the ratio of activity against MU-STAg/MU-TAg, the primary axis has data shown relative to WT (i.e., WT = 1), while the secondary axis denotes the absolute values of these ratios. All values are from the determination of activities at 37 °C, as shown in Table 1.

Upon comparison of our evolved GH101s with the GH101 sequence record it is evident that our introduced mutations are deviations from the consensus and are quite rare in Nature (Figure 5B). In particular, the high level of conservation of the residue equivalent to D1254 and the near complete loss of activity toward MU-TAg (Figure 4C) we observed upon its mutation suggests a crucial role for this residue in recognition of the TAg by most GH101s. However, more generally, throughout this campaign it seems that the improvements in activity against the STAg have come at the cost of activity to the TAg (Figure 5C). The respecialization of enzymes - such as *Sp*GH101 in this evolutionary trajectory - occurs often in directed evolution and speaks to the activity trade-offs that are inherent in evolution.^36,56,57^

During the preparation of this paper, a new study was released which describes a class of GH101s which seem to have a greater substrate scope than most GH101s.^58^ Comparison of active site structures between our evolved *Sp*GH101 variant, M3, and the sole characterized enzyme from this new class (POGase AS from an Actinomyces sp. (GenBank ID HHT41109.1)) shows a number of striking similarities. In particular, at positions equivalent to Q868, K1156, and E1253 we see very similar residues, which reduce negative charge and steric hindrance in the putative −3 subsite (Figure S8).

## Conclusions

The STAg constitutes ∼60% of the O-glycans present in human serum.^23^ While previous work had identified GH101s with modest activity toward this structure,^22,52^ poor catalytic activities made it impractical for most purposes. Through rapid exploration of sequence space by droplet-based microfluidics we were able to generate both greatly improved STAg-cleaving enzymes and those with inverted selectivity compared to the WT in just two rounds of directed evolution. Importantly, by miniaturizing the screen in pL-sized droplets, we were able to use a complex substrate for which the required syntheses would not be feasible on the scale required for plate-based screening of libraries of the size employed.

Our results highlight the value of applying a method for enzyme quantification to high-throughput assays. In this campaign, the expression level of the target enzyme within the pool of highly active clones isolated from the first round of microfluidic screening was substantially lower than that of the parent (>10-fold lower). While we cannot exclude the possibility that our improved mutants would have been found without the use of the mNeonGreen tag, the true extent of this improvement (>20-fold) was only determinable because of simultaneous measurement of expression levels. Based upon the data from M1, it is evident that the crucial E1253K mutation drives this decrease in soluble expression. This change in activity at the cost of expression/stability is relatively common in protein evolution and is a consequence of the often detrimental nature of mutations on protein stability.^40,59^ At a practical level, a number of directed evolution efforts have intentionally started with highly stable protein scaffolds as they are better able to accommodate such mutations.^60,61^ As shown in this work, a more generally applicable approach may be to instead use expression reporters to normalize for any decreases in enzyme expression/stability. Fluorescent protein fusions have long been used to engineer proteins for improved expression/stability^44,62,63^ but there are few examples of these being used in efforts looking to improve other aspects of a protein’s function.^38^ Such expression reporters may be of underappreciated utility as our results (along with precedent in the literature^38,39^ ) suggest that highly active variants can be overlooked due to losses in expression/stability. There is additional synergy within this approach as, if needed, one can further engineer the resultant highly active proteins to recover any losses in stability and/or expression by simply screening for improved expression using the same methods.^64^

Recent years have seen a number of advances in our understanding of the roles of glycans in health and disease driven by the application of new enzymatic tools. For example, an appreciation of the unique activities of O-glycopeptidases (sometimes referred to as mucinases) has opened new avenues in improving our understanding of mucin structures and functions.^65^ However, for many applications, it is not sufficient to rely upon the enzymes that are already available. Thus, to accommodate the needs of the research community, new approaches to enzyme discovery and engineering are required to help drive further advances.^49,66–68^ In this work, we have shown the utility of droplet microfluidics to reshape the active sites of GHs to better suit such demands. While it can be unwise to use non-natural substrates during screening campaigns since “you get what you screen for” it is important to note the observed correlation between the activity of *Sp*GH101 mutants against the chromogenic substrates used for screening and their equivalent structures in the context of glycoproteins. This correlation makes *Sp*GH101 an ideal candidate for further engineering to expand its substrate scope as improvements against readily synthesized and monitored chromogenic substrates could likely provide concomitant increases in activity against relevant glycoproteins. As further testament to the viability of this approach, the level of improvement we observe for *Sp*GH101 M2 against glycoproteins in this effort, is one of the largest improvements across all directed evolution campaigns of glycosidases when tested against their corresponding natural substrates.^66^ As shown in this work and in others,^22,26,69^ it seems that there are privileged scaffolds that are more capable of acting on glycoproteins and cell surfaces. It is these enzymes that would likely benefit most from directed evolution and/or other engineering approaches.

Overall, these results complement those on directed evolution of other classes of enzymes using a microfluidic screening strategy.^36,37^ The measured catalytic efficiencies of our improved enzymes against MU-STAg are well within the range of the majority of enzymes against their native substrates, highlighting the ability of ultrahigh-throughput screening to rapidly improve enzymatic activities.^70^ Although GHs are often highly specific for certain substrates, it seems likely that many GHs have low level promiscuous activities which are ready to be exploited given the opportunity.^71–73^ Droplet-based microfluidics is a powerful method for doing so. When applied, droplet-based microfluidics can unencumber the researcher from many of the limitations in library size and reagent usage that other methods suffer from—allowing massive libraries to be screened with complex, relevant substrates and to provide useful improvements to enzymatic properties.^29,36^

## Supplementary Material



## Notes

Additional experimental details, materials and methods (including characterization), and supplementary data referenced within the text (PDF)

## Abbreviations

epPCR: error-prone PCR
JB: Jericho Blue
JB-STAg: Jericho Blue sialyl T-antigen
MU-TAg: Methylumbelliferyl T-antigen, MU-STAg, methylumbelliferyl sialyl T-antigen
Neu5Ac: 5-acetylneuraminic acid
TAg: T-antigen
STAg: sialyl T-antigen
