# Supplementary material for: Reshaping of a Glycoside Hydrolase Active Site through Expression-Compensated Droplet-Based Microfluidic Screening Provides Useful Tools for Glycomics
Source: ACS Cent Sci. 2025 Sep 2;11(10):1993–2005. doi: 10.1021/acscentsci.5c01227 (PMC12550630; doi:10.1021/acscentsci.5c01227)
Supplement: Supplementary file 1 [file oc5c01227_si_001.pdf]

Supporting Information:

Reshaping of a Glycoside Hydrolase Active Site through Expression-Compensated Droplet-based Microfluidic Screening Provides Useful Tools for Glycomics

Jacob F. Wardman[1,2,3], Feng Liu[2,3], Saulius Vainauskas[4], Charlotte Olagnon[2,3], Teresa A. Howard[2,3], Yuqing Tian[2,3], Seyed A. Nasseri[2,3], Rajneesh K. Bains[2,3], Christopher H. Taron[4], Stephen G. Withers[1,2,3]*

[1] Department of Biochemistry and Molecular Biology, University of British Columbia, Vancouver, BC V6T 1Z3, Canada
[2] Michael Smith Laboratories, University of British Columbia, Vancouver, BC V6T 1Z4, Canada
[3] Department of Chemistry, University of British Columbia, Vancouver, BC V6T 1Z1, Canada
[4] New England Biolabs, Ipswich, Massachusetts 01938, United States

* to whom correspondence should be addressed: withers@chem.ubc.ca

This file includes:
Materials and methods
Tables S1-S3
Figure S1-S12
Supporting Information References

Materials and methods

General Methods

All buffers and reagents were purchased from Sigma-Aldrich or Thermo Fisher unless otherwise stated. Phusion DNA polymerase was used for all PCRs unless otherwise noted. All plasmid DNA minipreparations, PCR clean ups, and agarose gel extractions were carried out using kits from the GeneJET product line (Thermo Scientific). Carbenicillin (Carb) at 100 μg/mL was used throughout all culturing steps. All standard microtiter plates were from Corning, and all deep well plates were from Axygen.

Synthesis of glycan substrates

MU-TAg and MU-STAg were obtained from a previous study[1].

JB-STAg was synthesized enzymatically from JB α-*N*-acetylgalactosaminide (kindly provided by Dr. Hongming Chen) using the β1-3 galactosyltransferase CgtB and the α2-3 sialyltransferase Cst-I in a single pot reaction. The reaction mixture consisted of 5 mM JB α-*N*-acetylgalactosaminide, 7.5 mM UDP-Gal, 15 mM CMP-Neu5Ac, 10 mM $MgSO_4$, 0.125 mg/mL CgtB, 0.125 mg/mL Cst-I, and 0.250 mg/mL HUST6 in a final volume of 9.6 mL. Note that this does produce JB di-sialyl T-antigen, however this product was not used within this study and was removed during purification of JB-STAg. The reaction was left to incubate overnight at 37°C. The following day, an additional 0.5 equivalents of CMP-Neu5Ac and UDP-Gal were added to the reaction before additional incubation for 24 hours at 37°C. Proteins were then removed through use of Amicon Centrifugal filter columns (10 kDa MWCO). JB-STAg was separated from other JB-containing species via ion exchange chromatography using a gravity flow DEAE Sephadex A-25 anion exchange (40-120 μm particle size) column (Pharmacia Fine Chemicals) through

stepwise addition of different concentrations of ammonium acetate. Ammonium acetate concentrations ranged from 40 – 500 mM with JB-STAg eluting at 80 – 120 mM. The product was then desalted over Filmtec NF270-4040 membrane by washing with ddH2O. A final purification step of HILIC (Xbridge BEH Amide, 5 μm, 10 mm x 250 mm, Waters) was also performed. Product identity was confirmed by mass spectrometry. HRMS for $C_{35}H_{45}FN_2NaO_{23}$ (M+Na): calculated 903.2295, found 903.2289.

^{1}H, ^{13}C and 2D NMR spectra were obtained on Brucker Avance 600 MHz spectrometer with cryoprobe. The signals were assigned by H-H COSY and H-C HSQC experiments with linkages confirmed using HMBC spectrum.

^{1}H NMR (600 MHz, D_2O) δ 8.29 (s, 1H, ^{JB}H-4), 7.53 (dd, $J_{5,F}$ = 1.8 HZ, $J_{5,6}$ = 9.0, 1H, ^{JB}H-5), 7.33 (dd, $J_{5,6}$ = 9.0, $J_{6,F}$ = 6.9 Hz, 1H, ^{JB}H-6), 5.84 (d, $J_{1,2}$ = 3.5 Hz, 1H, ^{GalNAc}H-1), 5.12 (d, $J_{1,2}$ = 7.8 Hz, 1H, ^{Gal}H-1), 4.59 (dd, $J_{1,2}$ = 3.4 Hz, $J_{2,3}$ = 9.7 Hz, 1H, ^{GalNAc}H-2), 4.39-4.36 (m, 2H, ^{GalNAc}H-3 and ^{GalNAc}H-4), 4.18– 4.15 (m, 1H, ^{GalNAc}H-5), 4.13 (dd, $J_{2,3}$ = 9.8 Hz, $J_{3,4}$ = 3.1 Hz, 1H, ^{Gal}H-3), 3.99–3.97 (m, 1H, ^{Gal}H-4), 4.59 (ddd, J = 8.8, 6.3, 2.6 Hz, 1H, ^{Neu5Ac}H-8), 3.89-3.84 (m, 2H, ^{Neu5Ac}H-5 and ^{Neu5Ac}H-9a), 3.80 – 3.71 (m, 4H, 2x^{GalNAc}H-6 and 2x^{Gal}H-6), 3.71-3.63 (m, 4H, ^{Gal}H-5, ^{Neu5Ac}H-4, ^{Neu5Ac}H-6 and ^{Neu5Ac}H-9b), 3.63 – 3.58 (m, 2H, ^{Neu5Ac}H-7 and ^{Gal}H-2), 2.78 (dd, $J_{3a,3b}$ = 12.4 Hz, $J_{3a,4}$ = 4.6 Hz, 1H, ^{Neu5Ac}H-3a), 2.06 (s, 3H, ^{Gal}Ac), 2.05 (s, 3H, ^{Neu5Ac}Ac),1.82 (dd, $J_{3a,3b}$ = 12.4 Hz, $J_{3b,4}$ = 11.8 Hz, 1H, ^{Neu5Ac}H-3b).

^{13}C NMR (151 MHz, D_2O) δ 177.8 (^{Gal}Ac), 176.7 (^{Neu5Ac}Ac), 176.8 (^{Neu5Ac}COOH), 163.1 (^{JB}C-2), 149.9 (d, $J_{C,F}$ = 7.7 Hz, ^{JB}C-7), 147.5 (^{JB}C-4), 145.7 (d, $J_{C,F}$ = 9.2 Hz, ^{JB}C-8a), 142.7 (d, $J_{C,F}$ = 249 Hz, ^{JB}C-8), 127.3 (d, $J_{C,F}$ = 4.2 Hz, ^{JB}C-5), 125.7 (^{JB}C-3), 118.1 (^{JB}C-4a), 117.3 (^{JB}C-6), 107.39 (^{Gal}C-1), 102.57 (^{Neu5Ac}C-2), 100.32 (^{GalNAc}C-1), 79.6 (^{GalNAc}C-3), 78.5 (^{Gal}C-3), 77.7 (^{Gal}C-5), 75.7 (^{Neu5Ac}C-6), 75.0 (^{GalNAc}C-5), 74.7 (^{Neu5Ac}C-8), 71.95 (^{Gal}C-2), 71.3 (m, 2xC, ^{GalNAc}C-4 and ^{Neu5Ac}C-4), 70.9 (^{Neu5Ac}C-7), 70.3 (^{Gal}C-4), 65.4 (^{Neu5Ac}C-9), 63.87 (^{Gal}C-6), 63.8 (^{GalNAc}C-6), 54.5 (^{Neu5Ac}C-5), 51.2 (^{GalNAc}C-2), 42.6 (^{Neu5Ac}C-3), 24.9 (^{Neu5Ac}Ac), 24.8 (^{Gal}Ac).

Cloning

mNeonGreen was amplified from a plasmid containing mNeonGreen using the primers mNeonGreen_SpGH101_Fwd and mNeonGreen_SpGH101_Rev. pCW SpGH101 Q868G (as obtained from a previous study[1]) was then amplified with the primers

SpGH101_mNeonGreen_Fwd and SpGH101_mNeonGreen_Rev (primer sequences shown in Table S3). The mNeonGreen insert was then ligated into pCW SpGH101 Q868G via PIPE cloning.

For creation of the epPCR library of *Sp*GH101 Q868G mutants, error prone PCR using DreamTaq DNA Polymerase (following the manufacturer's suggested conditions supplemented with 0.01 – 0.1 mM $MnCl_2$) was used to create mutant PCR products carrying *Sp*GH101 Q868G using the primers shown in Table S3. Note that only the 3 kb region of *Sp*GH101 (from amino acids 334-1404) corresponding to domains conserved in GH101s was mutated[1]. The inserts were then assembled via Gibson assembly into the pCW *Sp*GH101 Q868G mNeonGreen vector and transformed into E. cloni 10G Elite electrocompetent cells (Lucigen) which were then plated on LB + Carb. The cells were recovered by addition of 0.5X LB + 25% glycerol to the plates and scraping using a cell spreader. At the same time, the size of the library was determined by plating serial dilutions of the transformation mixture onto LB + Carb plates, and then counting colonies to determine the total number of transformants.

Site-directed mutagenesis was carried out using the M-PIPE protocol[2]. Each mutation (E548V, I574V, and E1050A) was introduced using the forward and reverse primer pairs noted in Table S3.

For the creation of the simultaneous site-saturation mutagenesis library, the mutants were generated by overlap extension PCR using the primers in Table S3. In brief, the genes were first amplified via PCR with primers carrying degenerate codons to generate fragments containing the mutated sites. To include the WT amino acid at position M616 within the library, an additional

PCR was run to generate the same product with the WT amino acid. The products were then mixed at a molar ratio of 1 WT M616 fragment: 6 TDK-mutagenized M616 fragment to provide a complete distribution of the desired amino acids. All PCR fragments were then combined in a single primerless PCR to generate the full-length insert. The full-length insert was isolated via gel extraction, and then further amplified with same primers used in the epPCR library. The insert was then assembled via Gibson assembly into the pCW *Sp*GH101 Q868G mNeonGreen vector and transformed into E. cloni 10G Elite electrocompetent cells (Lucigen) which were then plated on LB + Carb. The cells were recovered by addition of 0.5X LB + 25% glycerol to the plates and scraping using a cell spreader. At the same time, the size of the library was determined by plating serial dilutions of the transformation mixture onto LB + Carb plates, and then counting colonies to determine the total number of transformants.

For the creation of the library of backcrossed M4 mutants, the mutants were generated by overlap extension PCR using the primers in Table S3. Reactions were carried out as described above. However, in this case, WT and mutant PCR products for each fragment of the gene were mixed in a 1:1 ratio prior to overlap extension PCR to provide an equal mix of codons at each position. The Gibson assembly reaction was then transformed into electrocompetent Top10 cells and plated on LB + Carb. Individual colonies were then picked and arrayed into a 96-well plate for further screening as described in the section below entitled **Plate-based validation of screening results.**

Testing utility of mNeonGreen as a reporter for enzyme concentration

A 150 mL culture of *Sp*GH101 Q868G mNeonGreen was grown overnight at 37°C in auto-induction media with Carb. The following day, the cells were washed twice with 150 mL 1 X PBS pH 7.0, and then lysed by sonication (3 min pulse time; 5 sec pulse, 15 sec pause, 35% amplitude) on ice in 30 mL 1 X PBS pH 7.0 supplemented with 0.5 mg/mL lysozyme and 1X cOmplete EDTA-free proteinase inhibitor. The lysate was then centrifuged at 16,000 x g for 10 minutes at 4°C and 50 μL of clarified lysate was then pipetted into a black 96-well plate, which was incubated for 10 minutes at 37°C. mNeonGreen fluorescence was then determined by reads at EX: 490 nm, EM: 520 nm. Following this, 50 μL of pre-heated 20 μM MU TAg in 1X PBS pH 7.0 was added to each well. The release of MU was monitored by fluorescence at EX: 365 nm, EM: 450 nm over time. Observed mNeonGreen fluorescence was converted to protein concentration using a standard curve derived from the fluorescence of purified mNeonGreen protein.

Droplet generation and sorting

E. coli libraries were grown from glycerol stocks in 25 mL LB + Carb until OD600 ~ 0.5. An assay mixture was then made up containing 0.5 X PBS pH 7.0, 0.5 X LB supplemented with 100 μM JB STAg, Carb, and 50 μM IPTG. For the first round of screening (the epPCR library), cells were diluted into the assay mixture such that 0.5 cells were encapsulated in each droplet in the first round of sorting, 0.25 in the second and third round, and 0.1 cells/droplet in the fourth round. In the second round of screening (the simultaneous site-saturation mutagenesis library), 0.8 cells were encapsulated in each droplet in the first round of sorting, and 0.1 cells/droplet in the second round of sorting. Note that in this second round of screening, IPTG was omitted from the assay mixture. As well, generally, decreasing droplet occupancy was used in order to decrease the occurrence of false positives during sorting and to improve sorting fidelity.

The droplets were produced by flowing the cell-containing assay mixture into the central inlet of a commercially available PDMS chip (DG-DMI-45 from Droplet Genomics) with HFE-7500 (3M) + 0.5% FluoSurf surfactant (Emulseo) being flowed into the second inlet. Droplet generation was controlled using an ElveFlow OB1 Mk3 pressure controller. The produced droplets had a diameter of 60-65 μm upon production. In the first round of screening, droplets were stored overnight at 37°C in screw cap tubes before sorting, while in the second round, droplets were stored at 30°C in screw cap tubes. Sorting was carried out using an On-Chip Sort droplet sorter with the 150 μm sorting chip in the first round of screening and an 80 μm sorting chip in the second round of screening. Gates were set based on forward and side scatter, and mNeonGreen (EX: 448 nm, EM: 543/22 nm) and Jericho Blue fluorescence (EX: 405 nm, EM: 445/20 nm). A sample gating strategy is shown in Figure S1.

Droplets were recovered as previously described[3] with the lysed droplets then being spread on plates of LB agar + Carb + 0.5% (w/v) glucose. The cells were then recovered by addition of 0.5X LB + 25% glycerol to the plates and scraping using a cell spreader. Where appropriate the cells were then grown and sorted again.

Plate-based validation of screening results

A master plate was generated by picking and arraying individual colonies into sterile 96-well plates containing LB + 10% glycerol + Carb. Cells were allowed to grow overnight at 37°C. For the first round of screening (the epPCR library), the master plate was used to inoculate a 96-well deep well plate with 1000 μL LB + Carb + 50 μM IPTG. The cultures within the deep well

plate were then grown and induced overnight at 37°C with shaking. For the second round of screening (epPCR library), the cultures within the deep well plate were first grown in LB + Carb in the absence of IPTG for 4 hours at 37°C with shaking before addition of IPTG to 50 μM, and overnight expression at 25°C with shaking. The following day, the cultures were pelleted for 5 minutes at 2900 x g in a swinging bucket centrifuge. The supernatant was then decanted and the cells resuspended in 500 μL 1 X PBS pH 7.0. The cells were then pelleted as before, the supernatant removed, and the cells resuspended in PBS pH 7.0 again. The cells were then lysed by resuspension in 200 μL of 1X PBS pH 7.0 + 0.5% Triton X-100 + 0.5 mg/mL hen egg white lysozyme. Lysis was allowed to proceed for 3-6 hours at room temperature with gentle shaking. The cells were then pelleted 10 minutes at 2900 x g in a swinging bucket centrifuge. 50 μL of clarified cell lysate was then transferred from each well into a black half-area 96-well plate. For the first round of screening, the plate was incubated for 10 minutes at 37°C. mNeonGreen fluorescence was then determined by reads at EX: 490 nm, EM: 520 nm. Following this, 50 μL of pre-heated 50 μM STAg JB in 1X PBS pH 7.0 was added to each well. The release of Jericho Blue was then monitored by fluorescence (EX: 390 nm, EM: 450 nm) over time. In the second round of screening and in the screen of backcrossed M4 mutants, incubations and reactions were carried out at room temperature instead of 37°C and only 50 μL of 25 μM JB-STAg was added. As well, assay plates prepared in the same manner were also separately assayed against MU-TAg by addition of 50 μL of 25 μM MU-TAg in 1X PBS pH 7.0 to each well.

Released glycan labelling and uHPLC-MS assays

Glycan release assays were performed largely as previously described[1]. All assays were carried out with 100 nM enzyme with incubations at 37°C in a heated water bath. All reagents were pre-heated to 37°C prior to starting the reaction. At each time point, reactions were stopped by addition of 3 volumes of ice-cold EtOH. These stopped reactions were then stored at -20°C until all time points had been collected. Labelling and analysis was then performed with the same equipment as previously described[1]. The HPLC program used is as follows: 0–4 min, 11% acetonitrile/89% H_2O; 4–7 min, 11-40.2% acetonitrile/ 89–59.8% H_2O; 7.1–11 min, 95% acetonitrile/5% H_2O; 11.1–16.1 min 11% acetonitrile/89% H_2O. STAg product was monitored at m/z ratios of 761.2 and 761.3 in negative ion mode.

GH101 Sequence Analysis

All GH101 sequences as of January 19, 2023 were downloaded from the CAZy database as GenBank identifiers[4]. The corresponding sequences were then retrieved from GenBank[5]. Sequences were aligned in ClustalOmega using the default parameters via the EMBL-EBI interface[6,7]. Sequence logos were created using WebLogo[8].

Protein expression, purification, and kinetic characterization

The expression and purification of *Sp*GH101 variants were carried out as previously described[1].

Substrate depletion kinetics were performed as previously described[1,9]. In brief, under conditions where [S] << K_M, the equation for v_0 simplifies to:

$$v_0 = k_{kcat}[E][S]/K_M$$

And so, the pseudo-first order rate constant ($[E]k_{cat}/K_M$) can be determined by fitting of the reaction time course to a first order decay with the following equation:

$$[S]_t = [S]_0 e^{-k_{obs}t} = [S]_0 e^{-[E]k_{cat}/K_M t}$$

Division of the observed first order rate constant by [E] thus provides k_{cat}/K_M. Example substrate depletion plots are shown in Figure S9 and S10.

Determination of Michaelis-Menten kinetics was also carried out as previously described[1]. Michaelis-Menten plots are shown in Figure S11.

The thermal stability of each enzyme was determined by diluting the enzymes to 0.017 mg/mL in PBS pH 7.0. They were then incubated for 10 minutes at temperatures ranging from 25 – 80°C in a thermocycler before being held at 25°C. They were then diluted in PBS (1- to 100-fold depending on the activity of the enzyme) and assayed against 12.5 µM MU-TAg. Thermal stability plots are provided in Figure S12.

Table S1. Activities of triple mutant variants of M1 against MU-STAg at 37°C. All values are shown as mean ± standard error of the mean (n = 3-7). An error of 5% was assumed for all values unless determined to be higher experimentally. [b] The GH101s assayed did not display saturation kinetics towards the substrate under the conditions employed and so the substrate depletion method was used to determine k_{cat}/K_M as previously described[9].

Mutations	k_{cat}/K_M (s^{-1} mM^{-1})	Fold-Change in k_{cat}/K_M over WT
E548V Q868G E1253K	35.1 ± 1.8 [b]	121
I574V Q868G E1253K	32.2 ± 1.8 [b]	111
Q868G E1050A E1253K	31.9 ± 1.6 [b]	110

Table S2. Thermostability of *Sp*GH101 variants characterized in this work. Enzyme variants were incubated for 10 minutes at a range of different temperatures from 25 – 80°C. After cooling to room temperature, they were then assayed for activity against MU-TAg. Values indicate average ± standard error of the fit.

Enzyme	T_{50}^{10} (°C)
**WT**	46.8 ± 0.5
**Q868G**	45.9 ± 0.2
**M1**	42 ± 0.4
**M2**	43 ± 0.9
**M3**	42.9 ± 0.4
**M4**	43 ± 1

Table S3. Primers used in this work. Bolded sequences denote sites that were subjected to either site-directed mutagenesis or site-saturation mutagenesis,

Primer Name	Sequence	Purpose
**SpGH101_mNeonGreen_Fwd**	GGGATCCGGATAAGTCGACAAGCTTATCGATG	Fusing mNeonGreen to the C-terminus of SpGH101 via PIPE cloning.
**SpGH101_mNeonGreen_Rev**	CCCTTGCTCACTGTCACAACAAGTTTCACATC	
**mNeonGreen_into_SPOG_Fwd**	CTTGTTGTGACAGTGAGCAAGGGCGAG	
**mNeonGreen_into_SPOG_Rev**	GCTTGTCGACTTATCCGGATCCCTTGTACAG	
**SpGH101_334_fwd**	GAAAAAGAAACAGGTCCTGAAGTTGATGATAGCAAGGTGACTTATGACACG	Amplifying SpGH101 for library creation via Gibson assembly. Primers allow for mutagenesis of the region from amino acids 334-1404 of SpGH101.
**SpGH101_1404_rev**	CCTGTTAGGGTAATTTCTTCGATTTGAAGATTATCCATCATGAAGTCGTTATA	
**pcw_SpGH101_334_rev**	CGTGTCATAAGTCACCTTGCTATCATCAACTTCAGGACCTGTTTCTTTTTC	Amplifying the vector for SpGH101 cloning.
**pcw_SpGH101_1404_fwd**	TATAACGACTTCATGATGGATAATCTTCAAATCGAAGAAATTACCCTAACAGG	
**SpGH101_E548V_fwd**	GTAGGAATCCACAGCTCT**gtA**TGGCAATGGGAAAAAG	Site directed mutagenesis of SpGH101 to introduce the E548V mutation.
**SpGH101_E548V_rev**	**tac**AGAGCTGTGGATTCCTACATAGTTGGCATTTCCG	
**SpGH101_I574V_fwd**	CTTCCAAGTGCTAAGGTTGTT**gTC**ACTGAAGATGCCAATGCAGACAAGAAAG	Site directed mutagenesis of SpGH101 to introduce the I574V mutation.
**SpGH101_I574V_rev**	**gac**AACAACCTTAGCACTTGGAAGTTCCTTCGTG	
**SpGH101_E1050A_fwd**	CGTTCGAAACAAACCAATCCT**GcA** ATGTCATGGAGTGAAGGCA TG	Site directed mutagenesis of SpGH101 to introduce the E1050A mutation.
**SpGH101_E1050A_rev**	**tgc**AGGATTGGTTTGTTTCGAACGATAGAG aacg	
**SpGH101_M616X_F618X_fwd**	GGATATCACAGCTTACCGTATCGCG **TDK** AAC **TDK** GGTTCTCAAGCACAAAACCCATTCC	Simultaneous site-saturation of M616 and F618 with hydrophobic residues.
**SpGH101_M616WT_F618X_fwd**	GGATATCACAGCTTACCGTATCGCG ATG AAC **TDK** GGTTCTCAAGCACAAAACCCATTCC	
**SpGH101_616618_rev**	CGCGATACGGTAAGCTGTGATATCC	
**SpGH101_K1156X_fwd**	CAATAAGTCTCTCGCCCTCAACTATGTA **NNS** GCCTATGCCCACAATACACGTC	Simultaneous site-saturation of K1156 with all possible amino acid residues.
**SpGH101_K1156_rev**	TACATAGTTGAGGGCGAGAGACTTATTG	
**SpGH101_D1254X_fwd**	GGTGGTGTCGAAGGTGTCaAA **NNS** AACCGCACTCACTTGTCTG	

**SpGH101_D1254_rev**	TTtGACACCTTCGACACCACC	Simultaneous site-saturation of D1254 with all possible amino acid residues.
**SpGH101_M616F_fwd**	GGATATCACAGCTTACCGTATCGCG**TTT**AAC**TTT**GGTTCTCAAGCACAAAACCCATTCC	Shuffling different mutations to see effect of mutations on the activities of M4. Note that these fragments were subsequently assembled by overlap extension PCR using the SpGH101_334_fwd and SpGH101_1404_rev primers.
**SpGH101_M616WT_fwd**	GGATATCACAGCTTACCGTATCGCGATGAAC**TTT**GGTTCTCAAGCACAAAACCCATTCC	
**SpGH101_K1156L_fwd**	CAATAAGTCTCTCGCCCTCAACTATGTA**TTG**GCCTATGCCCACAATACACGTC	
**SpGH101_K1156_wt_fwd**	CAATAAGTCTCTCGCCCTCAACTATGTA**AAA**GCCTATGCCCACAATACACGTC	
**SpGH101_D1254N_fwd**	GGTGGTGTCGAAGGTGTCaAA**AAC**AACCGCACTCACTTGTCTG	
**SpGH101_D1254_WT_fwd**	GGTGGTGTCGAAGGTGTCaAA**GAC**AACCGCACTCACTTGTCTG	

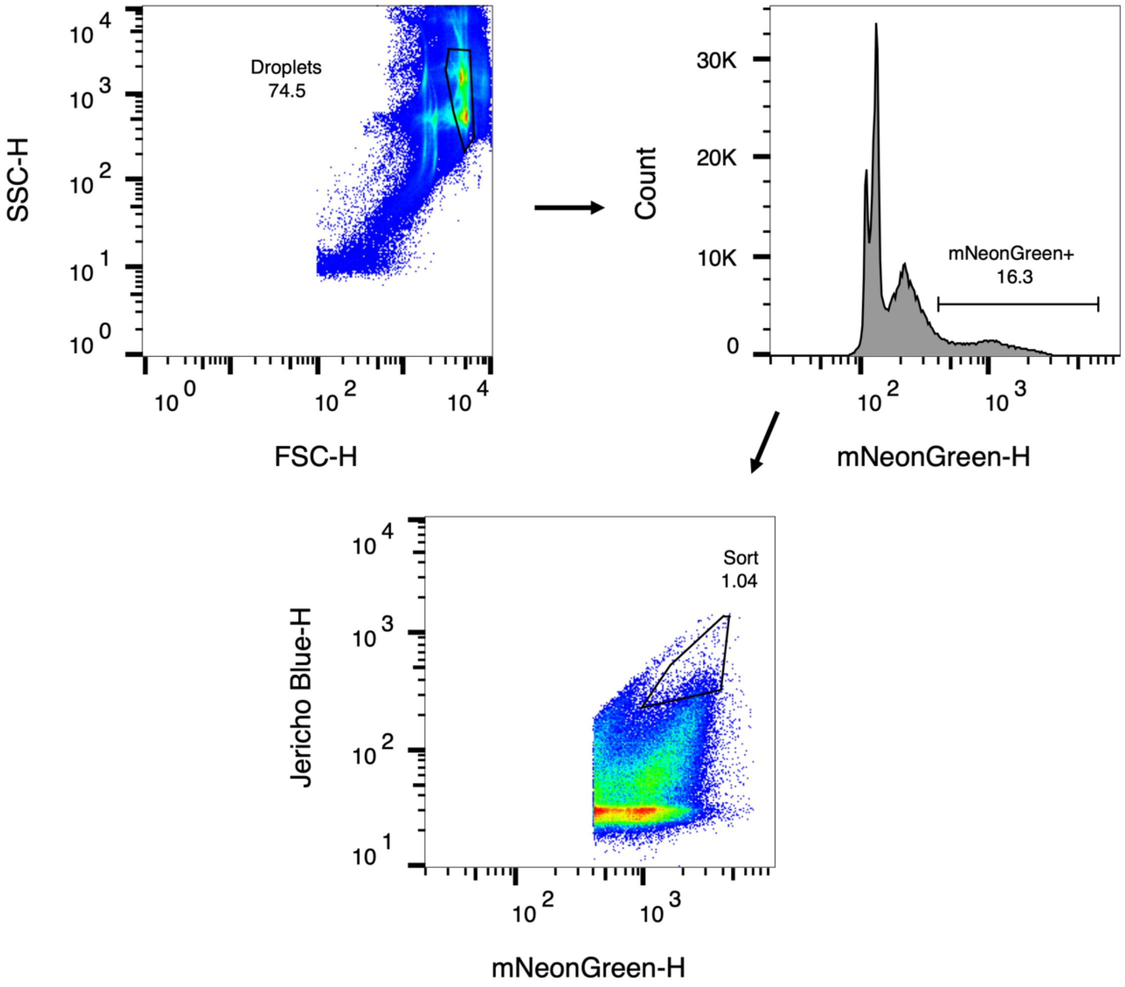

Figure S1. Sample gating strategy for enrichment of droplets containing highly active JB-STAg cleaving GH101 variants.

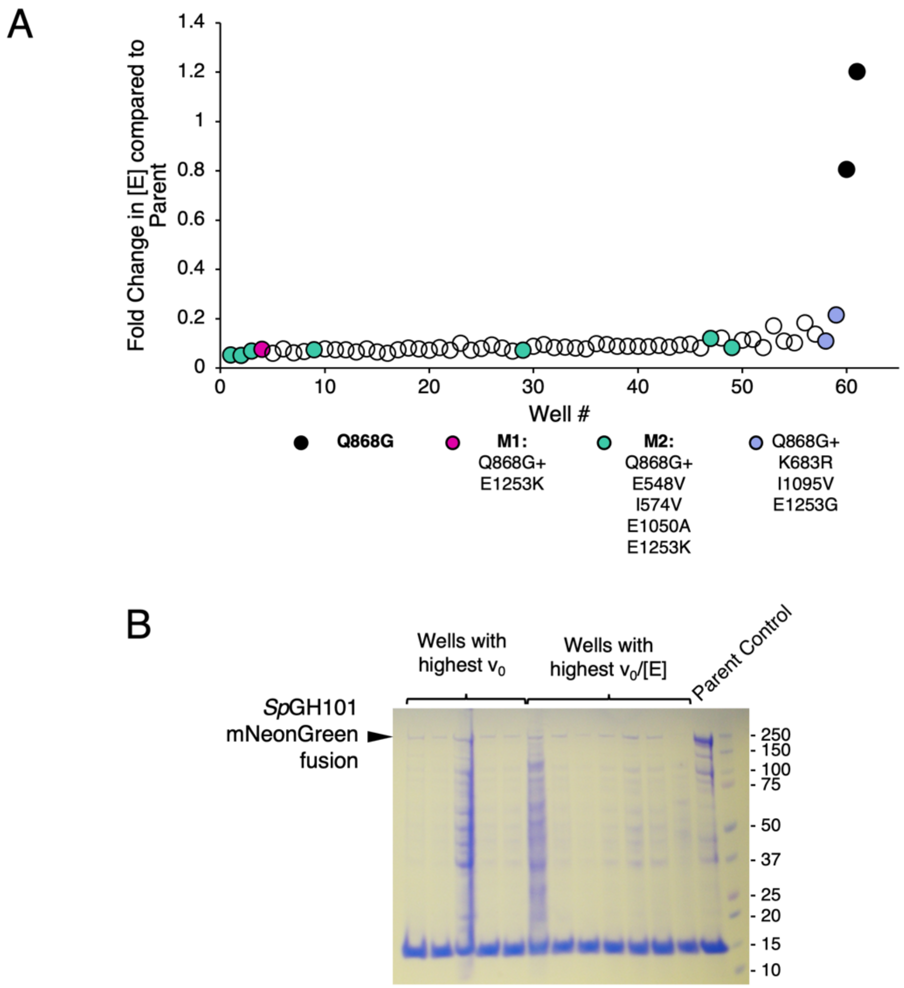

Figure S2. Enzyme concentrations during plate-based validation of an epPCR library screened for improved STAg-hydrolyzing *Sp*GH101 variants. **A**, The total amount of enzyme within the clarified lysate from the plate-based screen was measured using the mNeonGreen fluorescence of the mNeonGreen-tagged *Sp*GH101 variants. **B**, A selection of wells were then further analyzed by SDS-PAGE with subsequent visualization by Coomassie staining.

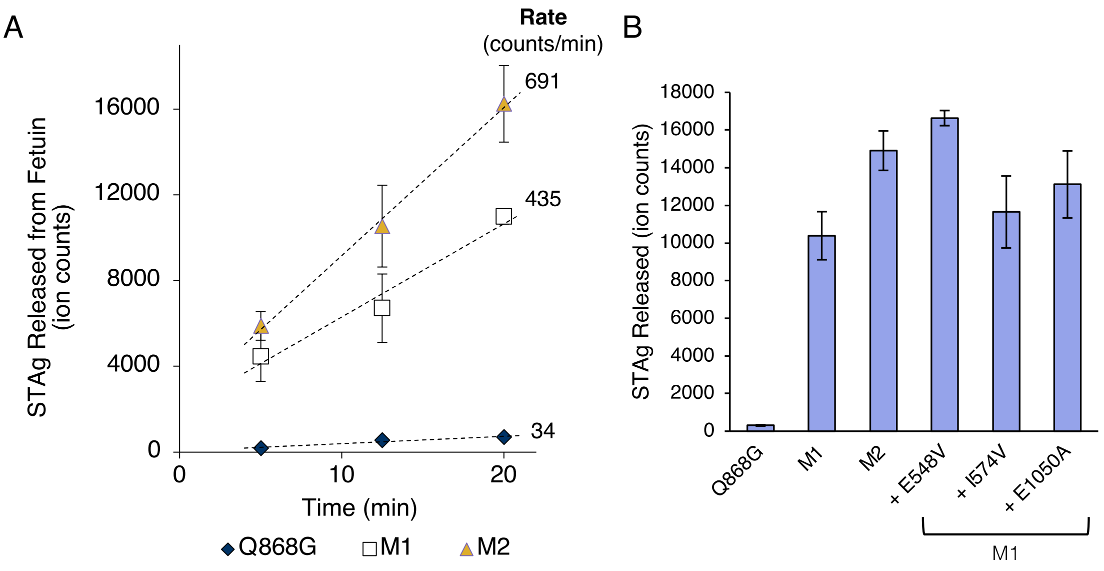

Figure S3. STAg hydrolase activity of SpGH101 variants against the model glycoprotein fetuin. A, Activity of *Sp*GH101 Q868G, M1, and M2 against fetuin as determined by the uHPLC-MS assay. B, STAg release by all *Sp*GH101 Q868G variants as measured after a 30 minute reaction. The peak corresponding to the STAg cleavage product in each sample was identified by comparison to a standard prior to integration as done previously[1]. Data points indicate mean ± standard error of the mean: n=4 for M1 in panel A and n=2 for all other data points. The dashed line in **A** indicates the linear fits for the different sets of data points.

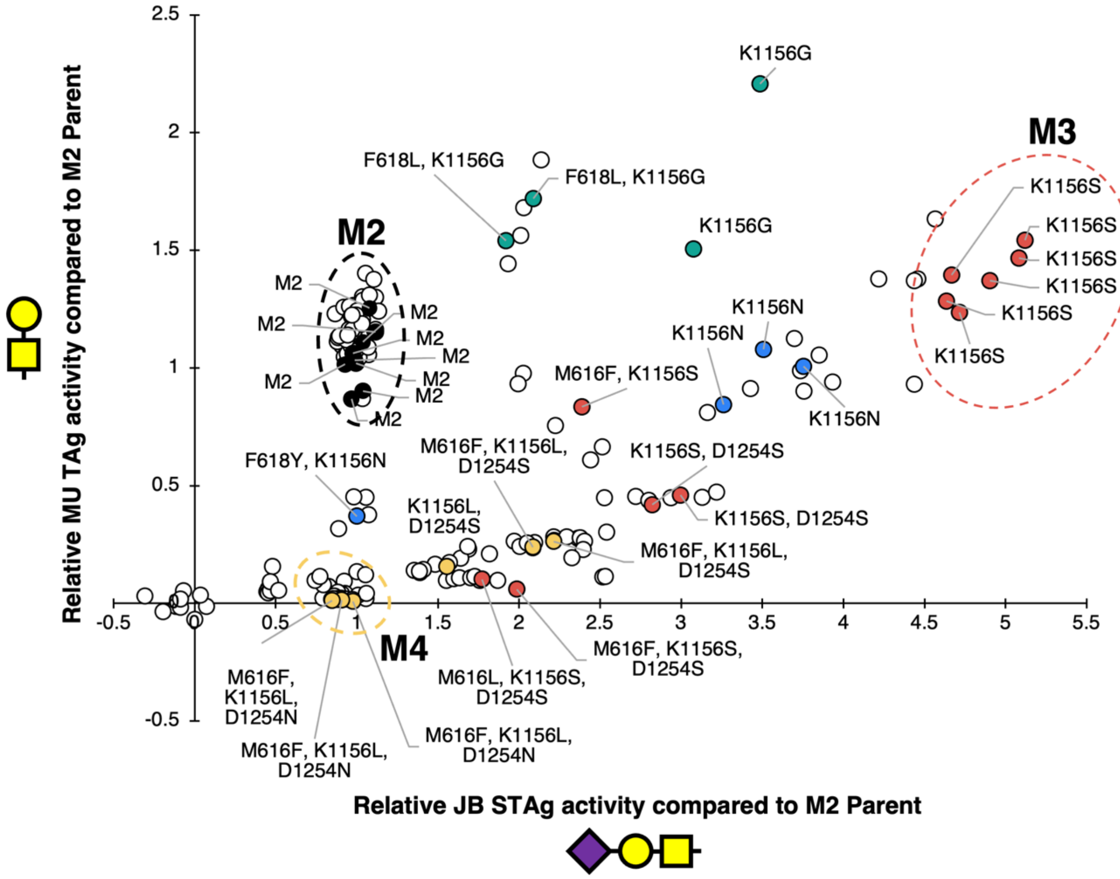

Figure S4. Activity of *Sp*GH101 variants derived from a simultaneous site-saturation mutagenesis library. Note that this is the same data as shown in Figure 4B but with all sequenced data points labeled. Data points are colored based upon the identity of the amino acid at position 1156 as mutation of this position seems to provide large changes in activity and position 1156 was mutated in all sequenced variants.

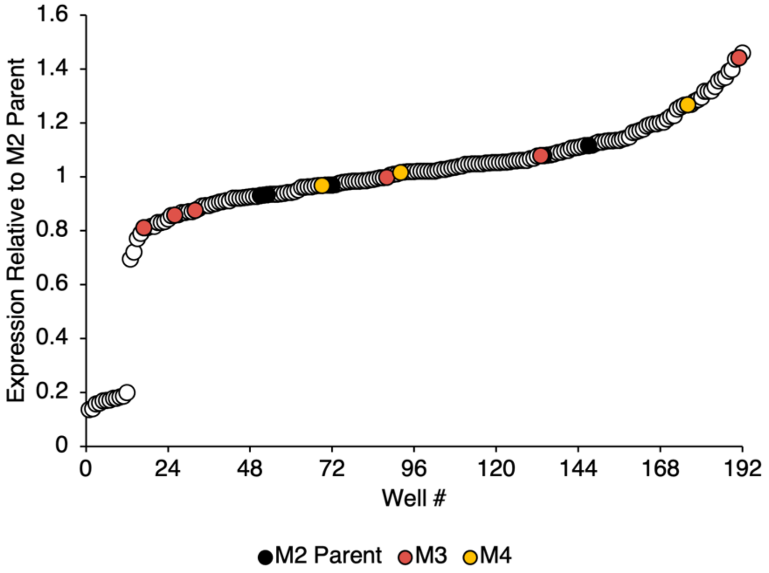

Figure S5. Enzyme concentrations during plate-based validation of a simultaneous site saturation library screened for improved STAg hydrolyzing *Sp*GH101 variants as determined by mNeonGreen fluorescence. Note that the clones with extremely low observed mNeonGreen fluorescence did not provide substantial enzymatic activity against MU-TAg or JB-STAg.

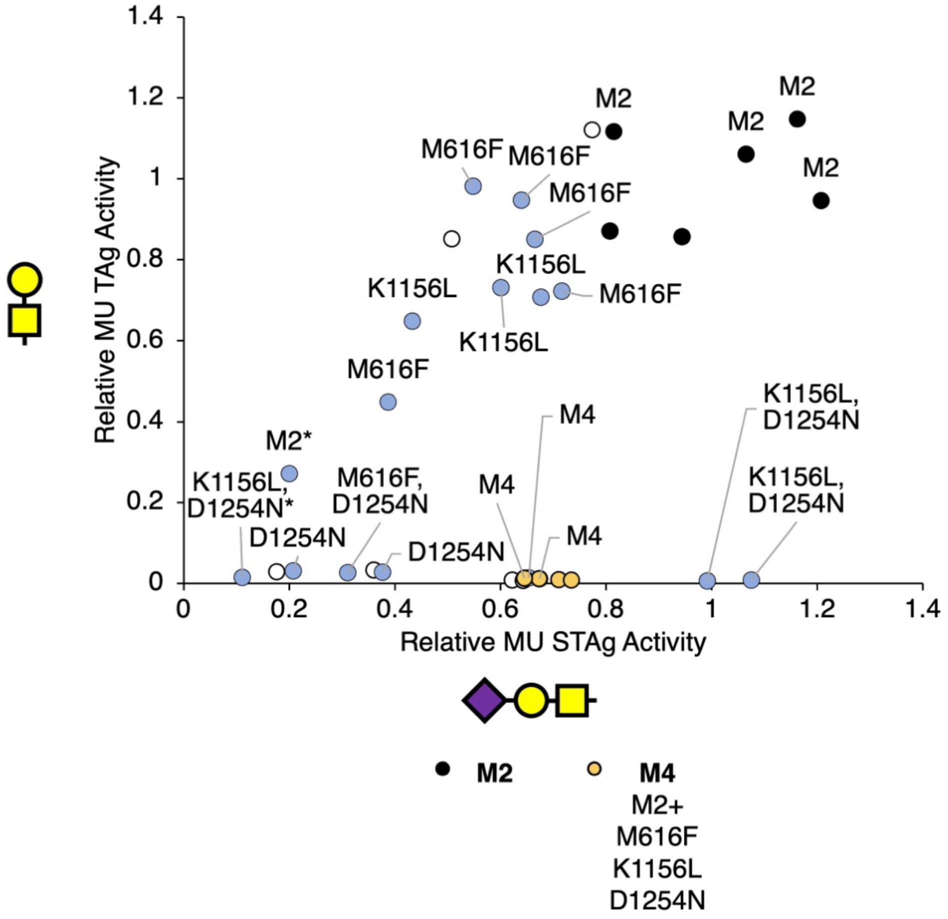

Figure S6. Screen of shuffled M4 library to determine effects of different mutations. The new mutations within M4 (M616F, K1156L, D1253N) were shuffled with the wild type residues using M2 as a template. The resulting library was then screened for activity against MU-TAg and MU-STAg. A number of different mutants were then sequenced to determine the underlying mutations. The data points with asterisks contains the indicated mutations as well as other mutations outside of the desired set of mutations.

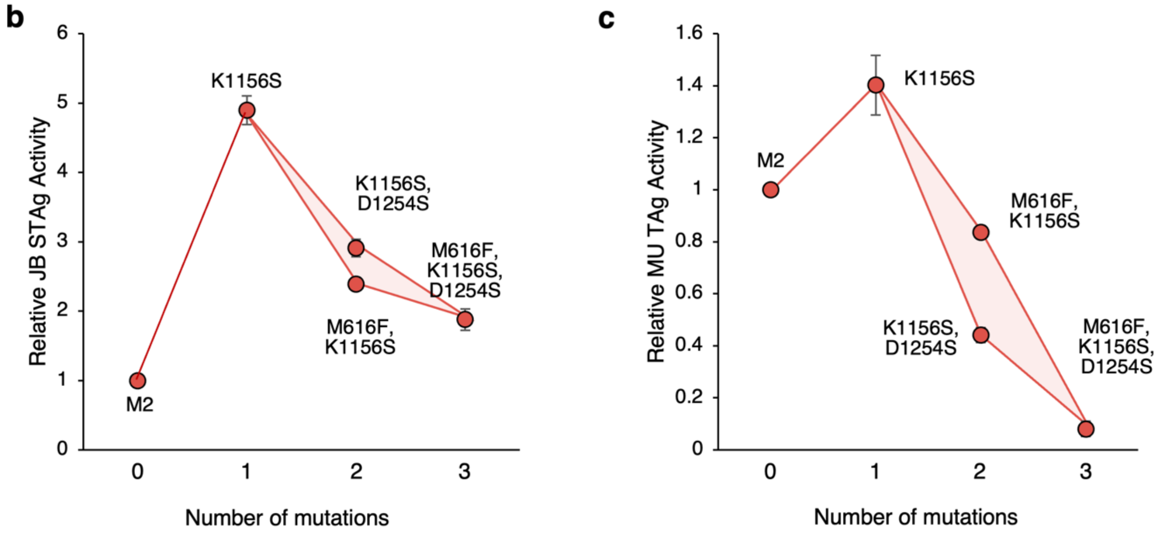

Figure S7. Combinations of mutations different from those in M4 also produce *Sp*GH101 variants with high selectivity for STAg over TAg. Note that this is a re-plot of data points shown in Figure 4B and Figure S4. n=1-6 depending upon number of sequenced variants for each mutant.

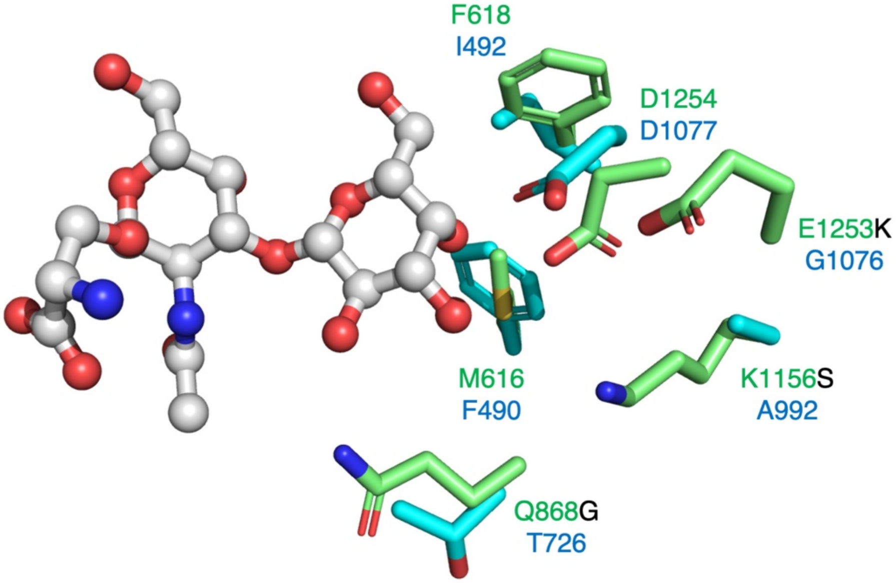

Figure S8. The evolved *Sp*GH101 variant M3 shows similar active site residues as the natively broad acting GH101, POGase AS. The structure of *Sp*GH101 TIGR4 co-crystallized with the serinyl T-antigen (shown in green) (PDB ID: 5A58)[10,11] was overlaid with an ColabFold[12] structure of POGase AS from *Streptomyces sp.* (shown in blue). Mutations to *Sp*GH101 found in variant M3 are shown in black. POGase AS is a recently discovered GH101 with natively high activity against the STAg among other structures[13].

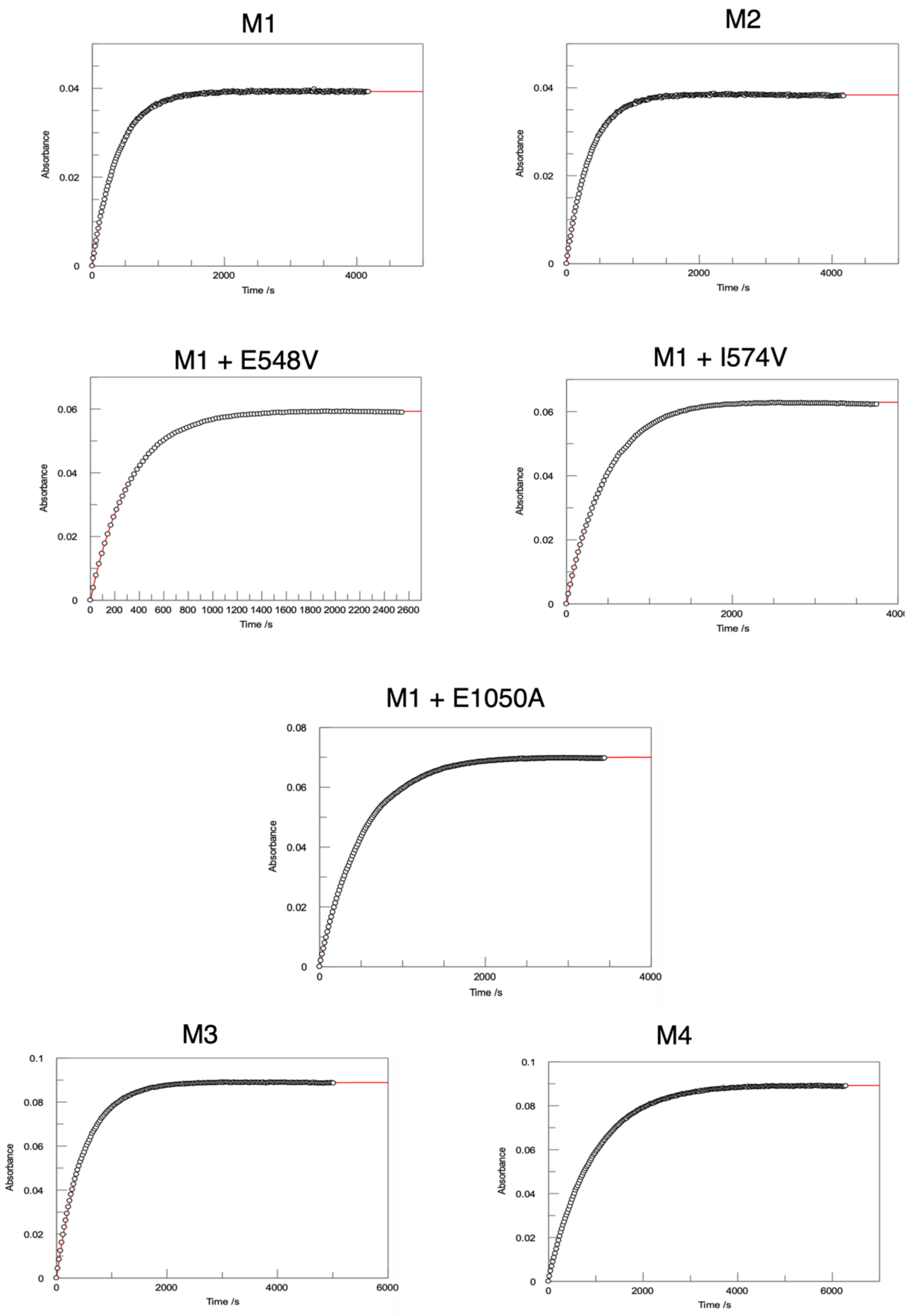

Figure S9. Substrate depletion plots for the activity of *Sp*GH101 variants against MU-STAg at 37°C. Plots are representative of 3 or 4 technical replicates.

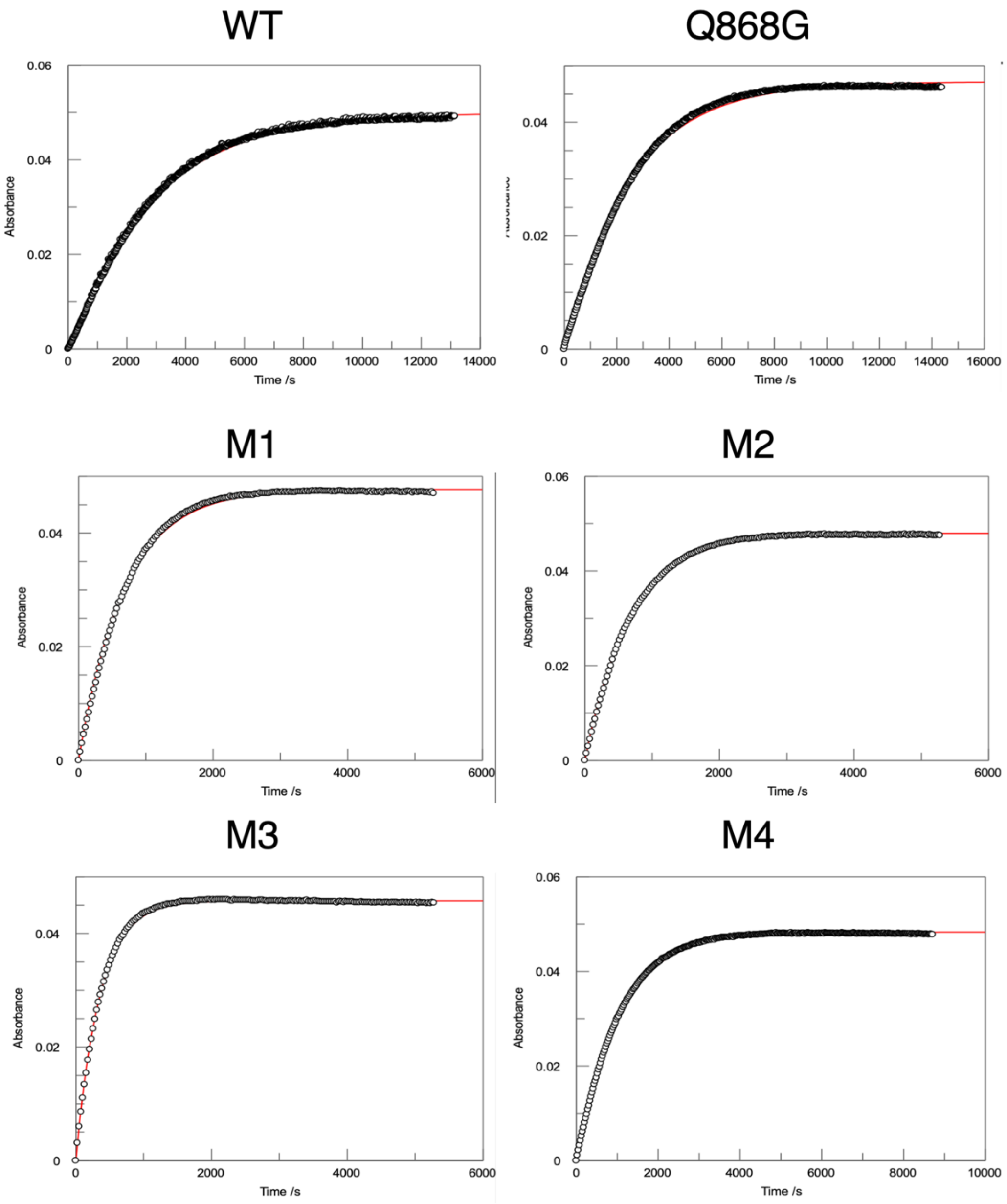

Figure S10. Substrate depletion plots for the activity of *Sp*GH101 variants against MU-STAg at room temperature. Plots are representative of 3 technical replicates.

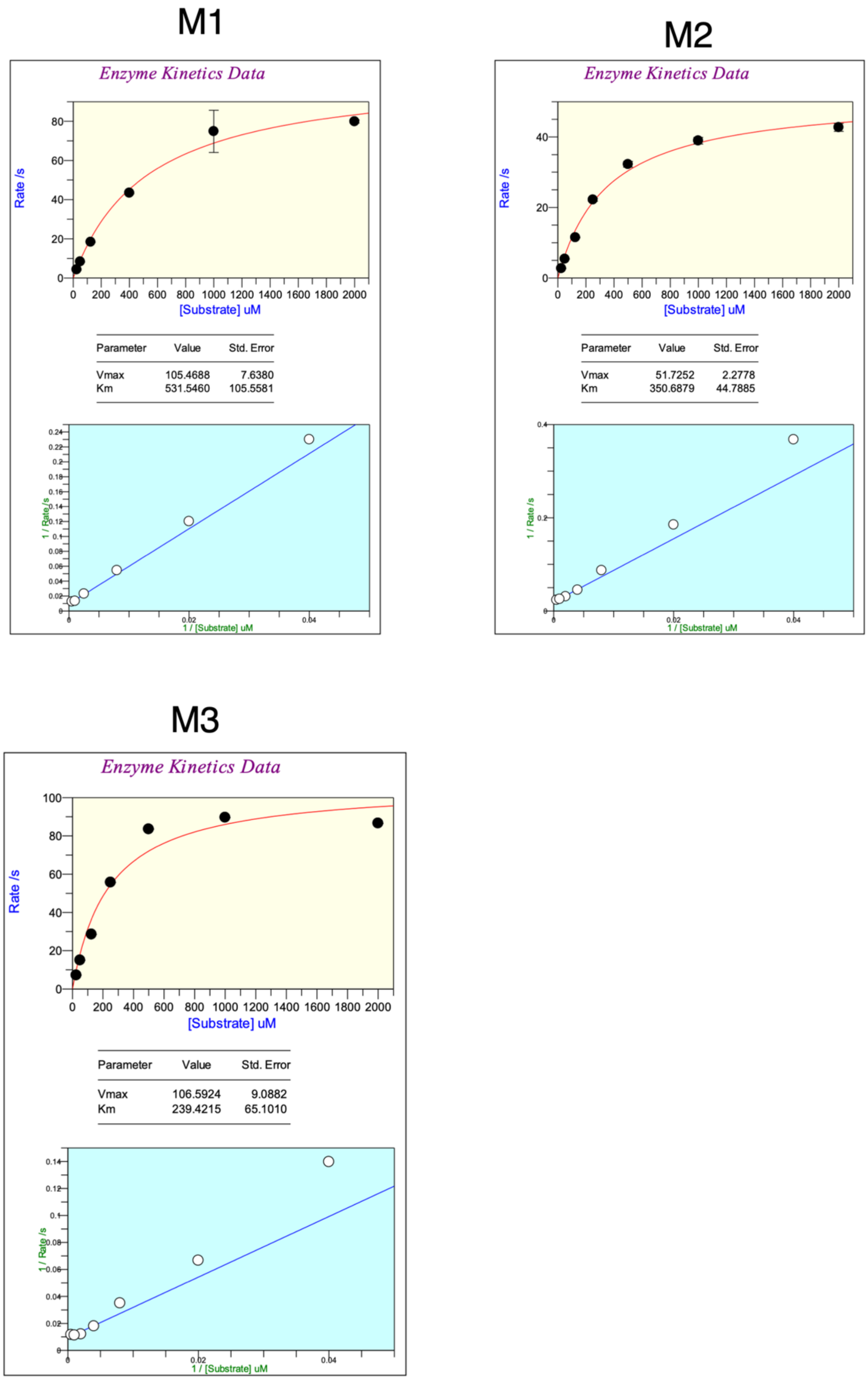

Figure S11. Michaelis-Menten kinetics of *Sp*GH101 variants against MU-TAg.

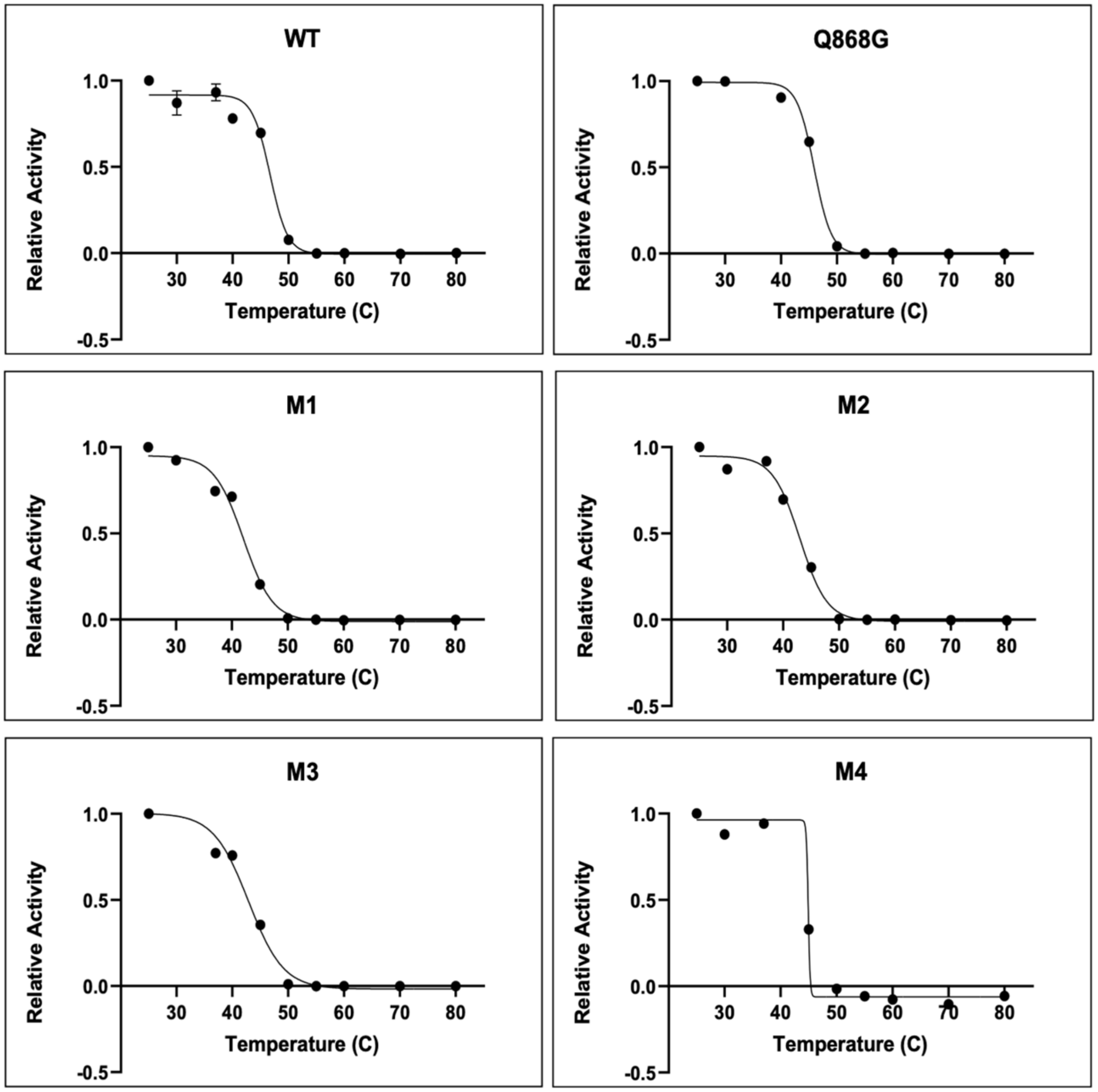

Figure S12. Thermal stability plots for the different *Sp*GH101 variants surveyed in this study. Values for the fit of these plots are provided in Table S2.

Supporting Information References:

(1) Wardman, J. F.; Rahfeld, P.; Liu, F.; Morgan-Lang, C.; Sim, L.; Hallam, S. J.; Withers, S. G. Discovery and Development of Promiscuous O-Glycan Hydrolases for Removal of Intact Sialyl T-Antigen. *ACS Chemical Biology* **2021**, *16* (10), 2004–2015. https://doi.org/10.1021/acschembio.1c00316.

(2) Klock, H. E.; Lesley, S. A. The Polymerase Incomplete Primer Extension (PIPE) Method Applied to High-Throughput Cloning and Site-Directed Mutagenesis. In *Methods in Molecular Biology: High Throughput Protein Expression and Purificiation*; 2009; pp 91–103.

(3) Olagnon, C.; Wardman, J. F.; Liu, F.; Chen, H.-M.; Moon, H.; Nasseri, S. A.; Seale, D.; Rahfeld, P.; Hallam, S. J.; Kizhakkedathu, J. N.; Withers, S. G. Ultrahigh-Throughput Single Emulsion Droplet Screening for the Discovery of New B Antigen Cleaving Enzymes. *ACS Catal.* **2024**, 12884–12894. https://doi.org/10.1021/acscatal.4c02165.

(4) Lombard, V.; Golaconda Ramulu, H.; Drula, E.; Coutinho, P. M.; Henrissat, B.; Ramulu, H. G.; Drula, E.; Coutinho, P. M.; Henrissat, B. The Carbohydrate-Active Enzymes Database (CAZy) in 2013. *Nucleic Acids Research* **2014**, *42* (D1), 490–495. https://doi.org/10.1093/nar/gkt1178.

(5) Benson, D. A.; Cavanaugh, M.; Clark, K.; Karsch-Mizrachi, I.; Lipman, D. J.; Ostell, J.; Sayers, E. W. GenBank. *Nucleic Acids Research* **2012**, *41* (D1), D36–D42. https://doi.org/10.1093/nar/gks1195.

(6) Madeira, F.; Madhusoodanan, N.; Lee, J.; Eusebi, A.; Niewielska, A.; Tivey, A. R. N.; Lopez, R.; Butcher, S. The EMBL-EBI Job Dispatcher Sequence Analysis Tools Framework in 2024. *Nucleic Acids Research* **2024**, *52* (W1), W521–W525. https://doi.org/10.1093/nar/gkae241.

(7) Sievers, F.; Higgins, D. G. Clustal Omega for Making Accurate Alignments of Many Protein Sequences. *Protein Science* **2018**, *27* (1), 135–145. https://doi.org/10.1002/pro.3290.

(8) Crooks, G. E.; Hon, G.; Chandonia, J.-M.; Brenner, S. E. WebLogo: A Sequence Logo Generator. *Genome Research* **2004**, *14* (6), 1188–1190.

(9) Vocadlo, D. J.; Wicki, J.; Rupitz, K.; Withers, S. G. Mechanism of Thermoanaerobacterium Saccharolyticum β-Xylosidase: Kinetic Studies. *Biochemistry* **2002**, *41* (31), 9727–9735. https://doi.org/10.1021/bi020077v.

(10) Gregg, K. J.; Suits, M. D. L.; Deng, L.; Vocadlo, D. J.; Boraston, A. B. Structural Analysis of a Family 101 Glycoside Hydrolase in Complex with Carbohydrates Reveals Insights into Its Mechanism. *Journal of Biological Chemistry* **2015**, *290* (42), 25657–25669. https://doi.org/10.1074/jbc.M115.680470.

(11) Berman, H. M.; Westbrook, J.; Feng, Z.; Gilliland, G.; Bhat, T. N.; Weissig, H.; Shindyalov, I. N.; Bourne, P. E. The Protein Data Bank. *Nucleic acids research* **2000**, *28* (1), 235–242. https://doi.org/10.1093/nar/28.1.235.

(12) Mirdita, M.; Schütze, K.; Moriwaki, Y.; Heo, L.; Ovchinnikov, S.; Steinegger, M. ColabFold: Making Protein Folding Accessible to All. *Nat Methods* **2022**, *19* (6), 679–682. https://doi.org/10.1038/s41592-022-01488-1.

(13) Zhou, L.; Ortega-Rodriguez, U.; Flores, M. J.; Matsumoto, Y.; Bettinger, J. Q.; Wu, W. W.; Zhang, Y.; Kim, S.-R.; Biel, T. G.; Pritts, J. D.; Shen, R.-F.; Rao, V. A.; Ju, T. Dual

Functional POGases from Bacteria Encompassing Broader O-Glycanase and Adhesin Activities. *Nat Commun* **2025**, *16* (1), 1960. https://doi.org/10.1038/s41467-025-57143-8.
